# SlVQ15 recruits SlWRKY30IIc to link with jasmonate pathway in regulating tomato defence against root‐knot nematodes

**DOI:** 10.1111/pbi.14493

**Published:** 2024-11-05

**Authors:** Huang Huang, Xuechun Ma, Lulu Sun, Yingying Wang, Jilin Ma, Yihan Hong, Mingjie Zhao, Wenchao Zhao, Rui Yang, Susheng Song, Shaohui Wang

**Affiliations:** ^1^ Plant Science and Technology College Beijing University of Agriculture Beijing China; ^2^ Beijing Key Laboratory for Agricultural Application and New Technique Beijing University of Agriculture Beijing China; ^3^ College of Life Sciences Capital Normal University Beijing China

**Keywords:** defence, *M. incognita*, tomato, SlJAZs, VQ proteins, WRKY transcription factors

## Abstract

Tomato is one of the most economically important vegetable crops in the world and has been seriously affected by the devastating agricultural pest root‐knot nematodes (RKNs). Current understanding of tomato resistance to RKNs is quite limited. VQ motif‐containing family proteins are plant‐specific regulators; however, whether and how tomato VQs regulate resistance to RKNs is unknown. Here, we found that SlVQ15 recruited SlWRKY30IIc to coordinately control tomato defence against the RKN *Meloidogyne incognita* without affecting plant growth and productivity. The jasmonate (JA)‐ZIM domain (JAZ) repressors of the phytohormone JAs signalling associated and interfered with the interaction of SlVQ15 and SlWRKY30IIc. In turn, SlWRKY30IIc bound to *SlJAZs* promoters and cooperated with SlVQ15 to repress their expression, whereas this inhibitory effect was antagonized by SlJAZ5, forming a feedback regulatory mechanism. Moreover, *SlWRKY30IIc* expression was directly regulated by SlMYC2, a SlJAZ‐interacting negative regulator of resistance to RKNs. In conclusion, our findings revealed that a regulatory circuit of SlVQ15‐SlWRKY30IIc and the JA pathway fine‐tunes tomato defence against the RKN *M. incognita*, and provided candidate genes and clues with great potential for crop improvement.

## Introduction

Tomato (*Solanum lycopersicum*), one of the most widely cultivated vegetable crops worldwide, plays an essential role in global agricultural production. Root‐knot nematodes (RKNs, *Meloidogyne* spp.), soil‐borne biotrophic parasites with more than 3000 host species, are recognized as the most harmful and dangerous plant pathogenic nematodes (Abad *et al*., 2003). RKNs seriously threaten global tomato and agricultural production, causing approximately US$77 billion in losses annually (Castagnone‐Sereno *et al*., 2013; Jones *et al*., 2013; Ralmi *et al*., 2016).

The second‐stage RKN juveniles (J2s) infect tomato roots and induce 6–8 protoxylem cells to establish feeding sites called giant cells (Rutter *et al*., 2022). Giant cells and their surrounding cells form root‐knots (galls), which prevent tomato growth and development, and even cause plant death (Favery *et al*., 2016; Jones *et al*., 2013; Siddique and Grundler, 2018). J2 RKNs, which feed on giant cells, develop into males or females (Rutter *et al*., 2022). Females produce numerous eggs, and after hatching, they cause reinfection of the host plants (Rutter *et al*., 2022). Moreover, RKN infection provides favourable conditions for pathogen invasion to form complex diseases, increasing the difficulty of disease control (McSorleyR, 2011).

The control of RKNs is very difficult because of their high and fast reproduction rate (Trudgill and Blok, 2001). Chemical nematicides are considered the most useful method worldwide for reducing RKN disease (Abad *et al*., 2008; Collange *et al*., 2011). However, their applications with high cost and toxicity to environment have been severely restricted and increasingly withdrawn (Collange *et al*., 2011; Pulavarty *et al*., 2021). Safe, economical, and effective controls are required. The utilization of host resistance is considered a more effective and sustainable strategy to decrease the loss caused by RKNs. However, the knowledge on these is very limited. Therefore, characterization of the genetic determinants and molecular mechanism of plant resistance to RKNs is important for crop improvement and agricultural development, including tomato.

The phytohormones jasmonates (JAs) modulate tomato defence against RKNs (Cooper *et al*., 2005; Wang *et al*., 2019). The primary JA receptor Coronatine Insensitive 1 (COI1) and the JA‐ZIM domain (JAZ) repressors constitute COI1‐JAZ coreceptors to perceive JA signals, which triggers JAZs degradation and activates JA responses (Hu *et al*., 2023; Sheard *et al*., 2010; Wasternack and Song, 2017; Xie *et al*., 1998). The SlJAZ‐interacting transcription factor (TF) SlMYC2 integrates JA, abscisic acid (ABA), and strigolactone (SL) signals, and represses defence against *Meloidogyne incognita* (Xu *et al*., 2019). The SlJAZ‐associated TF SlMYB57, together with SlMYB108 and SlMYB112, regulates the balance of lateral root development and resistance to *M. incognita* (Zhao *et al*., 2023). The TF SlWRKY45 interacts with most SlJAZs, inhibits JA biosynthesis, and attenuates tomato resistance to *M. incognita* (Huang *et al*., 2022a). The COP9 signalosome subunits SlCSN4 and SlCSN5 interact with SlJAZ2 and enhance tomato resistance to *M. incognita* (Shang *et al*., 2019).

VQ motif (FxxhVQxhTG)‐containing proteins play vital roles in diverse processes via interacting with their partners (Jing and Lin, 2015). Arabidopsis VQ protein AtJAV1 interacts with AtWRKY51 and AtJAZ8 to repress JA biosynthesis for defence against herbivores (Yan *et al*., 2018). VQ‐containing AtSIB1/2 modulates AtWRKY33 to mediate resistance to the necrotrophic pathogen *Botrytis cinerea* (Lai *et al*., 2011). AtVQ18/26 associates with and acts as repressors of ABA‐Insensitive5 to control seed germination (Pan *et al*., 2018). AtVQ29 acts as a partner of Phytochrome‐Interacting Factor1 (PIF1) and inhibits seedling de‐etiolation (Li *et al*., 2014). Tomato contains 26 VQ proteins (Ding *et al*., 2019). However, their function and mechanism in resistance to RKNs are unknown.

Here, we discovered that SlVQ15 recruited SlWRKY30IIc to synergistically regulate tomato defence against the RKN *M. incognita* without reducing plant growth and productivity. SlJAZs associated with SlVQ15 and SlWRKY30IIc, and affected their interaction. In turn, SlWRKY30IIc repressed *SlJAZs* expression by binding to their promoters, which was enhanced by SlVQ15 but attenuated by SlJAZ5. Additionally, *SlWRKY30IIc* expression was inhibited by SlMYC2. These results revealed the molecular mechanism that the SlVQ15‐SlWRKY30IIc module controlled defence against the RKN *M. incognita* in tomato by linking with the JA pathway to form a feedback loop. These findings increase our understanding of tomato immunity and provide vital defence genes for the breeding of tomato and even other Solanaceae plants with resistance to RKNs.

## Results

### SlVQ15 plays a positive role in tomato resistance to the RKN *M. incognita*

**Figure 1:**
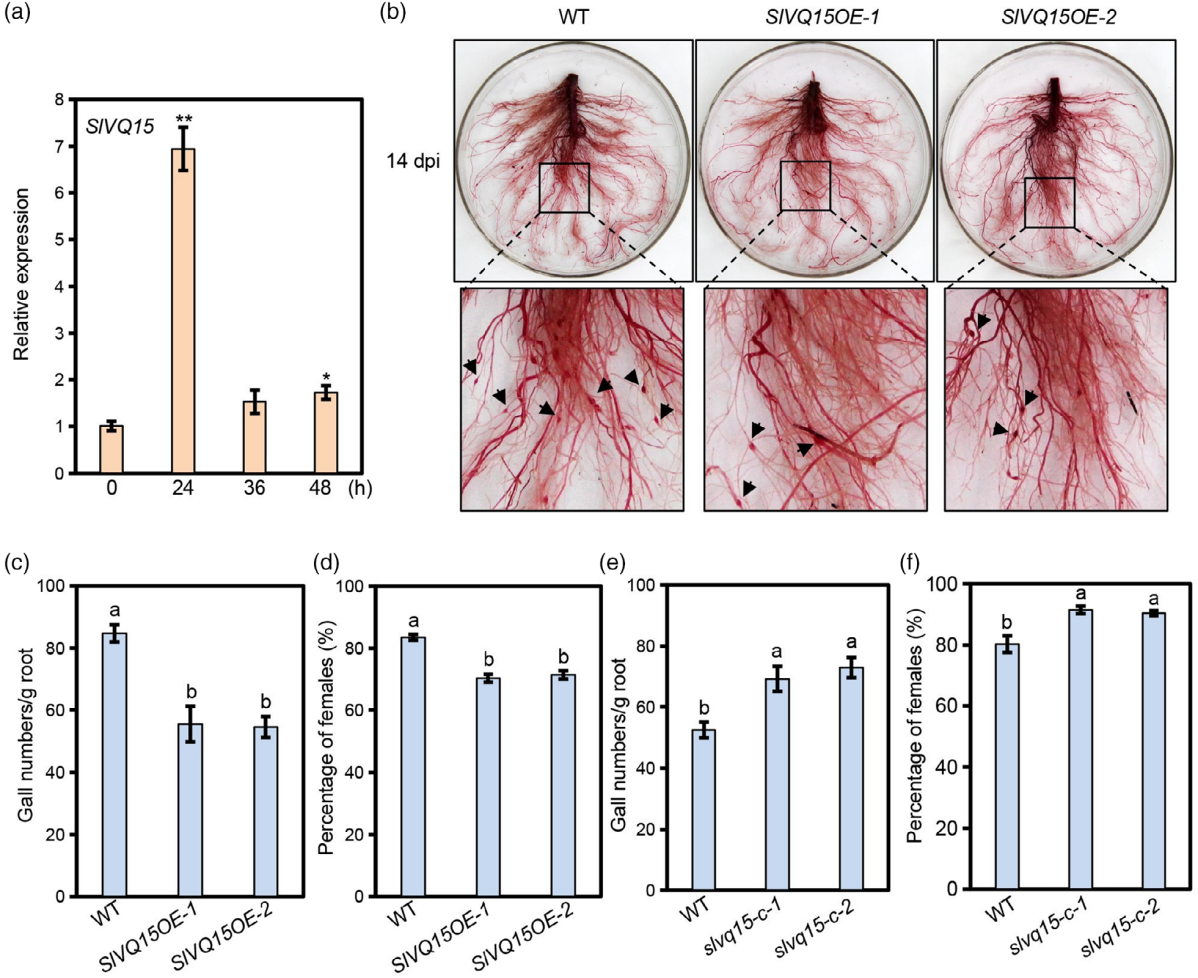
SlVQ15 acts as a positive regulator to control tomato defence against the RKN *Meloidogyne incognita*. (a) Relative expression levels of *SlVQ15* in roots of CM wild type (WT) with the RKN *Meloidogyne incognita* infection for 0, 24, 36, or 48 h. Values represent the means (±SE) of three independent biological replicates. Significant differences between the indicated time points and the control (0 h) were analysed by Student's *t*‐test (*P < 0.05, **P < 0.01). (b) Acid fuchsin staining of the roots from CM wild type and *SlVQ15*‐overexpressing plants (*SlVQ15OE‐1* and *SlVQ15OE‐2*) with *M. incognita* infection for 14 days. The black arrowheads indicate galls. (c–f) Gall numbers per gram of roots (c, e) and percentages of female nematodes (d, f) in the CM wild type and *SlVQ15*‐overexpressing plants (*SlVQ15OE‐1* and *SlVQ15OE‐2*) (c, d) or *slvq15* mutants (*slvq15‐c‐1* and *slvq15‐c‐2*) (e, f) at 14 days (c, e) or 35 days (d, f) after inoculation with *M. incognita*. Data represent the means (±SE) of 12 plants. The experiments were repeated three times with similar results. Different letters represent significant differences by one‐way ANOVA with Duncan's multiple range test (*P* < 0.05).

Given that the roles of VQ proteins in resistance to RKNs are unknown, we aimed to identify RKN infection‐responsive tomato VQs and investigate their function in defence against RKNs. Using quantitative real‐time PCR (qRT‐PCR) analysis, we found that the expression of several *SlVQ* genes (*SlVQ1‐6* and *9–15*) was differentially regulated after the RKN *M. incognita* infection and that *SlVQ15* and *SlVQ14* were the most highly induced among the detected *SlVQ* genes at 24 and 48 h after infection, respectively (Figure 1a, Figure S1). We next investigated the function of SlVQ15 in defence against *M. incognita*, as we have already obtained the overexpression lines and mutants of *SlVQ15* (Huang *et al*., 2022b).

We inoculated our previously generated *SlVQ15*‐overexpression lines (*SlVQ15OE‐1* and *SlVQ15OE‐2*) (Huang *et al*., 2022b) and the *Solanum lycopersicum* cv. Castlemart (CM) wild type with *M. incognita*. At 14 days after inoculation, the number of galls per gram of roots in the *SlVQ15*‐overexpression lines was obviously lower than that in the CM wild type (Figure 1b,c). We further analysed the percentage of females at 35 days after inoculation, another key parameter evaluating resistance to *M. incognita* (Xiang *et al*., 2020; Zhao *et al*., 2023), in the CM and *SlVQ15*‐overexpression lines. As shown in Figure 1d, the percentage of females was significantly reduced in the *SlVQ15*‐overexpression lines compared with that in the CM wild type, suggesting that overexpression of *SlVQ15* inhibited the development of *M. incognita*.

Having shown that *SlVQ15* overexpression enhanced resistance to *M. incognita*, we explored whether *slvq15* mutations could attenuate tomato defence using previously constructed *slvq15‐c‐1* and *slvq15‐c‐2* mutants (Huang *et al*., 2022b). Compared with the CM wild type, the *slvq15* mutants displayed a phenotype of increased susceptibility to *M. incognita*, as indicated by the fact that the gall numbers per gram of roots and the percentage of females were significantly increased (Figure 1e,f). Together, these results (Figure 1) demonstrated that SlVQ15 positively regulates resistance to *M. incognita* in tomato.

Furthermore, we found that the plant height, biomass, percentage of fruit set, and weight per mature fruit of the *SlVQ15*‐overexpression plants and *slvq15* mutants were similar to those of the CM wild type (Figure S2), suggesting that SlVQ15 may not affect morphological growth and fertility.

### SlVQ15 recruits and stabilizes SlWRKY30IIc

**Figure 2:**
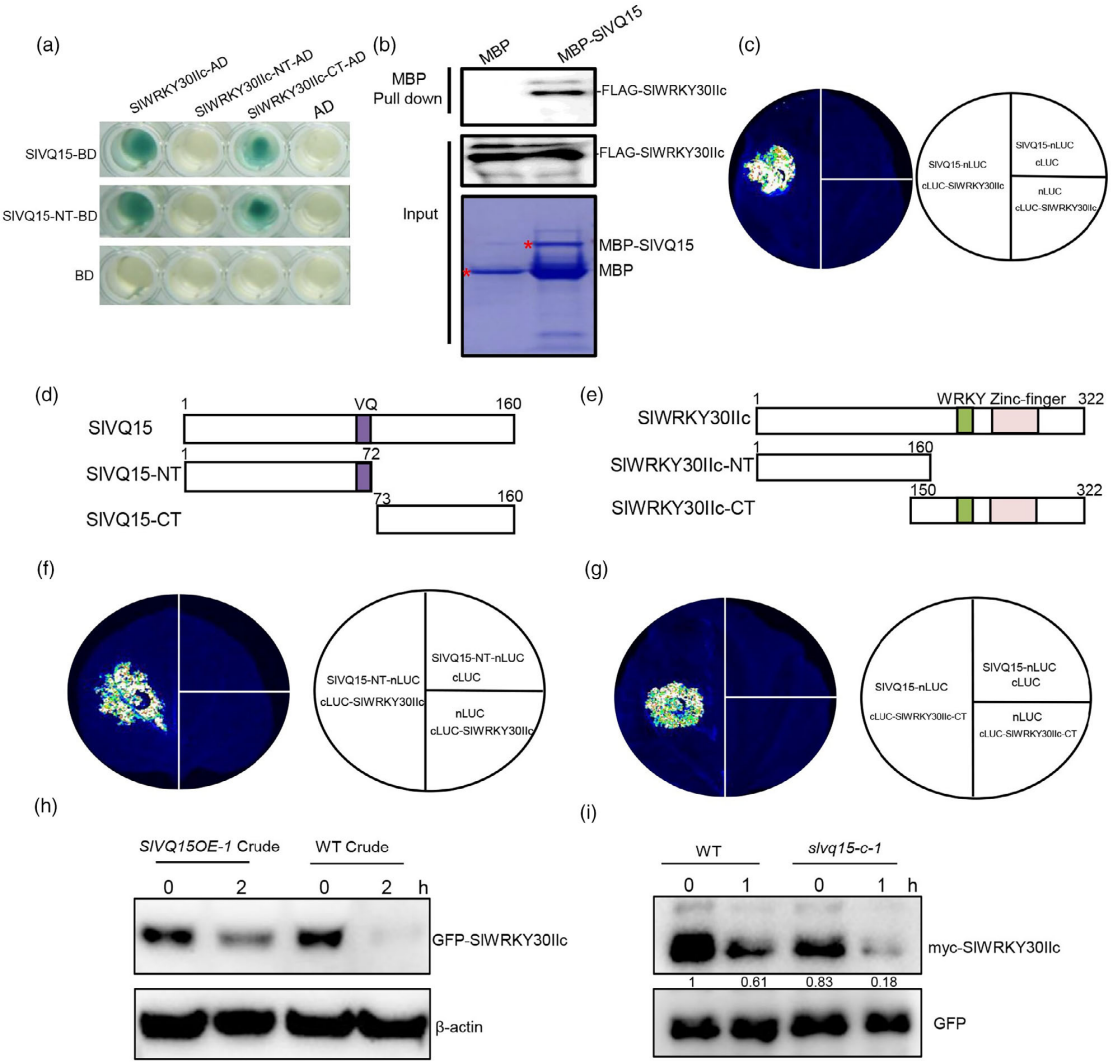
SlVQ15 physically interacts with SlWRKY30IIc to promote its stability. (a) Yeast two hybrid (Y2H) assays showing interactions of SlVQ15, SlWRKY30IIc, and their domains. SlVQ15, SlWRKY30IIc, and their related domains were fused with the DNA binding domain (BD) in pLexA or the activation domain (AD) in pB42AD. (b) Pull‐down assays to verify the interaction of SlVQ15 with SlWRKY30IIc. MBP‐SlVQ15 pulled down the transiently expressed FLAG‐SlWRKY30IIc, whereas MBP did not. (c) Firefly luciferase (LUC) complementation imaging (LCI) assays consistently demonstrated that SlVQ15 associates with SlWRKY30IIc. Luciferase activities were reconstituted with coexpression of N‐terminal part of LUC (nLUC)‐fused SlVQ15 (SlVQ15‐nLUC) and C‐terminal part of LUC (cLUC)‐fused SlWRY30IIc (cLUC‐SlWRKY30IIc). (d, e) Schematic diagrams display the domain constructs of SlVQ15 (d) and SlWRKY30IIc (e). The purple, green, and pink boxes, respectively, represent the VQ, WRKY, and Zinc‐finger domains, respectively. (f, g) LCI assays show that N‐terminal part of SlVQ15 interacts with SlWRKY30IIc (f), and that C‐terminal part of SlWRKY30IIc interacts with SlVQ15 (g). LUC activities were observed when SlVQ15‐NT‐nLUC and cLUC‐SlWRY30IIc (f), or SlVQ15‐nLUC and cLUC‐SlWRKY30IIc‐CT (g) were coexpressed. (h) SlVQ15 promoted the stability of SlWRKY30IIc protein. Purified GFP‐fused SlWRKY30IIc (GFP‐SlWRKY30IIc) was incubated with crude protein from the CM wild type (WT) or *SlVQ15*‐overexpressing lines (*SlVQ15OE‐1*) with 1 mm ATP for 0 and 2 h. The samples were detected by immunoblot analysis with anti‐GFP and anti‐β‐actin antibodies. (i) *In vivo* degradation assay showing that loss of SlVQ15 enhances SlWRKY30IIc protein degradation. myc‐SlWRKY30IIc plus GFP was cotransformed in tomato protoplasts isolated from the CM wild type (WT) and *slvq15* mutant (*slvq15‐c‐1*). At 16 h after transformation, protoplasts were treated with 50 μm protein synthesis inhibitor cycloheximide (CHX) for the indicated time points. GFP was used as an internal control. The samples were analysed by immunoblot analysis with anti‐myc and anti‐GFP antibodies. The numbers indicate the relative amounts of proteins normalized to GFP.

We employed yeast two hybrid (Y2H) screening assays to identify interaction proteins of SlVQ15. Of the 68 positive colonies (Table S1), fragments of a group IIc WRKY TF (Solyc07g056280) (Bai *et al*., 2018; Huang *et al*., 2012), named SlWRKY30IIc here, were captured four times (twice for aa 170–322 and twice for aa 148–322). Y2H assays confirmed that SlWRKY30IIc interacted with SlVQ15 (Figure 2a). We carried out a pull‐down assay and found that purified MBP‐fused SlVQ15 protein could pull down the transiently expressed FLAG‐fused SlWRKY30IIc, while purified MBP could not (Figure 2b). Moreover, firefly luciferase (LUC) complementation imaging (LCI) assays showed that LUC activity was strongly reconstituted with coexpression of the N‐terminal part of LUC (nLUC)‐fused SlVQ15 (SlVQ15‐nLUC) and the C‐terminal part of LUC (cLUC)‐fused SlWRKY30IIc (cLUC‐SlWRKY30IIc) in *Nicotiana benthamiana* leaves (Figure 2c), suggesting that SlVQ15 associates with SlWRKY30IIc in planta.

SlVQ15 was truncated into a N‐terminal part containing the VQ motif (SlVQ15‐NT) and a C‐terminal fragment (SlVQ15‐CT) (Figure 2d). SlWRKY30IIc was divided into a N‐terminal part (SlWRKY30IIc‐NT), and a C‐terminal fragment containing the WRKY and Zinc‐finger motif (SlWRKY30IIc‐CT) (Figure 2e). The Y2H results showed that SlVQ15 and SlVQ15‐NT interacted with SlWRKY30IIc and SlWRKY30IIc‐CT, but not with SlWRKY30IIc‐NT (Figure 2a). LCI assays consistently confirmed that the N‐terminal part of SlVQ15 and the C‐terminal part of SlWRKY30IIc are required for the SlVQ15‐SlWRKY30IIc interaction (Figure 2f,g).

We further truncated SlWRKY30IIc‐CT and SlVQ15‐NT into different domains to explore the minimal region required for their interaction (Figure S3a–d). The results showed that the SlWRKY30IIc‐CT3 with 82 amino acids of aa 170–251 was necessary for the interaction with SlVQ15 (Figure S3b). The short motif of SlVQ15‐NT2 (aa 42–72) was sufficient for the interaction with SlWRKY30IIc, whereas SlVQ15‐NT2 with a mutated VQ motif abolished their interaction, indicating that the VQ motif is essential for the SlWRKY30IIc‐SlVQ15 interaction (Figure S3d).

We next investigated whether SlWRKY30IIc protein stability is regulated by SlVQ15 to emphasize the significance of the SlVQ15‐SlWRKY30IIc interaction. We performed cell‐free degradation assays that GFP‐fused SlWRKY30IIc was transiently expressed in *N. benthamiana* leaves, purified by anti‐GFP beads, and added to total protein extracted from the CM wild type and *SlVQ15*‐overexpressing tomato lines. As shown in Figure 2h, GFP‐SlWRKY30IIc was more stable in the *SlVQ15*‐overexpressing crudes than in the CM wild‐type crudes.

Furthermore, we performed *in vivo* degradation assay using tomato protoplast system in which myc‐SlWRKY30IIc was transiently expressed in protoplasts from CM wild type and *slvq15* mutant. The results in Figure 2i showed that the protein abundance of myc‐SlWRKY30IIc at 0 h was reduced in the *slvq15* mutant protoplasts compared with the CM protoplasts, and that the degradation rate of myc‐SlWRKY30IIc in the *slvq15* mutant protoplasts was faster than that in the CM protoplasts. These findings suggested that SlVQ15 repressed SlWRKY30IIc degradation.

Taken together, these data (Figure 2) revealed that SlVQ15 interacts with and stabilizes SlWRKY30IIc.

### SlWRKY30IIc positively regulates resistance to *M. incognita*

We investigated the subcellular localization and expression pattern of SlWRKY30IIc. As shown in Figure S4a,b, SlWRKY30IIc was localized to the nucleus, and *SlWRKY30IIc* expression in roots was significantly induced by *M. incognita* infection. *SlWRKY30IIc* was expressed in roots, stems, leaves, flowers, and fruits, with notably high expression in roots (Figure S4c).

**Figure 3:**
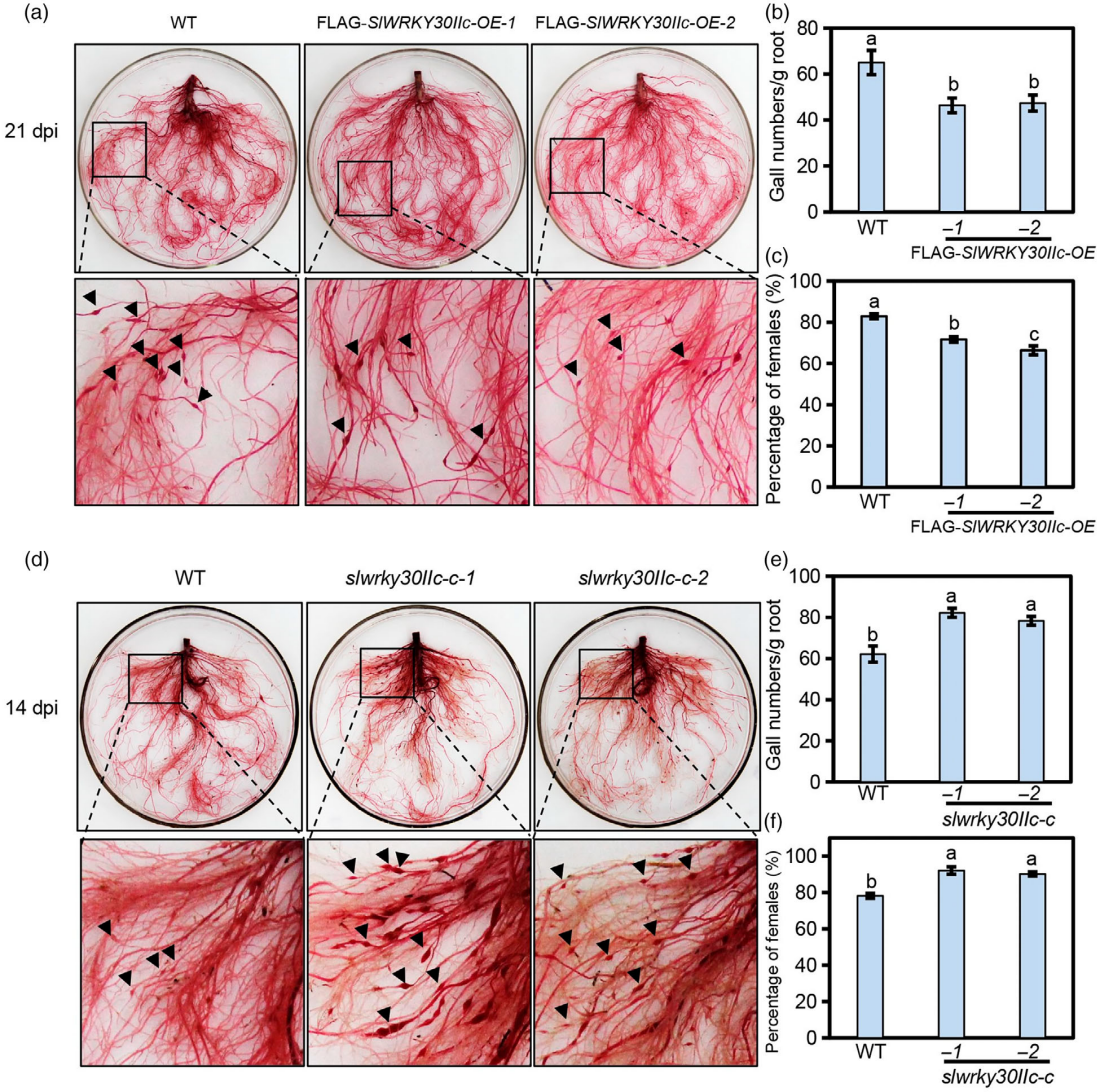
SlWRKY30IIc positively regulates resistance to the RKN *Meloidogyne incognita* in tomato. (a–f) Acid fuchsin staining of roots (a, d), gall numbers per gram of roots (b, e) and percentages of female nematodes (c, f) from the CM wild type and *SlWRKY30IIc*‐overexpressing plants (FLAG‐*SlWRKY30IIc‐OE‐1* and FLAG‐*SlWRKY30IIc‐OE‐2*) (a–c) or *slwrky30IIc* mutants (*slwrky30IIc‐c‐1* and *slwrky30IIc‐c‐2*) (d–f) after inoculation with *M. incognita* for 21 days (a, b), 35 days (c, f), or 14 days (d, e). The black arrowheads indicate galls. Data represent the means (±SE) of 12 plants. The experiments were repeated three times with similar results. Different letters represent significant differences by one‐way ANOVA with Duncan's multiple range test (*P* < 0.05).

To study the role of *SlWRKY30IIc* in resistance to RKNs, we generated *SlWRKY30IIc*‐overexpressing tomato lines in the CM background. Two transgenic lines, FLAG‐*SlWRKY30IIc‐OE‐1* and FLAG‐*SlWRKY30IIc‐OE‐2*, with approximately 11‐fold and 14‐fold of the CM wild‐type level of *SlWRKY30IIc*, were chosen as representatives for RKN infection assay (Figure S5). In comparison to the CM wild type, the FLAG‐*SlWRKY30IIc‐OE* plants presented lower gall numbers per gram of roots and percentage of females after inoculation with *M*. *incognita* (Figure 3a–c).

We generated *slwrky30IIc* mutants in the CM background via clustered regularly interspaced palindromic repeats (CRISPR)/CRISPR‐associated protein 9 (Cas9) gene‐editing technology with two target sequences in the first and second exons (Figure S6a). Two representative lines, namely, *slwrky30IIc‐c‐1* (2‐bp deletion in the first target, and 1‐bp insertion in the second target) and *slwrky30IIc‐c‐2* (1‐bp insertion in the first target) without mutations in potential off‐target sites, were used for *M. incognita* infection (Figure S6b,c). As shown in Figure 3d–f, the gall numbers per gram of roots at 14 days and percentage of females at 35 days after *M. incognita* infection in the *slwrky30IIc* mutants were greater than those in the CM wild type.

Moreover, we discovered that *SlWRKY30IIc*‐overexpression plants and *slwrky30IIc* mutants showed no significant difference from the CM wild type in terms of plant growth and fertility (Figure S7). These results suggested that SlWRKY30IIc positively controls defence against *M. incognita* and may have no obvious effect on tomato growth and development.

### SlVQ15 coordinates with SlWRKY30IIc to modulate defence against *M. incognita*

**Figure 4:**
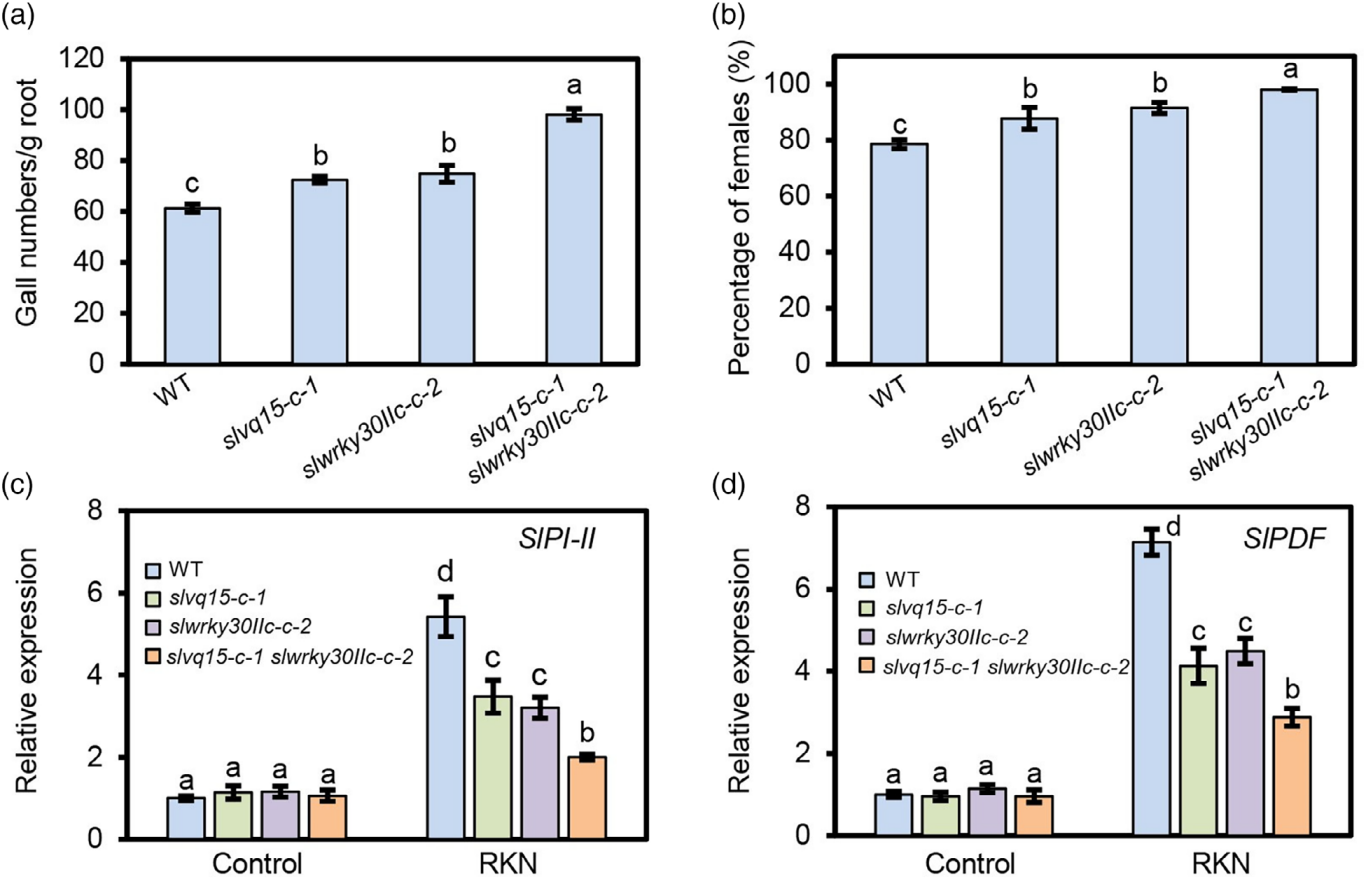
SlVQ15 and SlWRKY30IIc coordinately control resistance to the RKN *Meloidogyne incognita* in tomato. (a, b) Gall numbers per gram of roots (a) and percentage of female nematodes (b) of the CM wild type, *slvq15* mutants (*slvq15‐c‐1*), *slwrky30IIc* mutants (*slwrky30IIc‐c‐2*), and *slvq15 slwrky30IIc* mutants (*slvq15‐c‐1 slwrky30IIc‐c‐2*) at 14 days (a) and 35 days (b) after inoculation with *M. incognita*. Data represent the means (±SE) of 12 plants. The experiments were repeated three times with similar results. Different letters represent significant differences by one‐way ANOVA with Duncan's multiple range test (*P* < 0.05). (c, d) Relative expression levels of *SlPI‐II* (c) and *SlPDF* (d) in roots of the indicated plants without (control) or with *M. incognita* infection for 24 h. Data represent the means (±SE) of three independent biological replicates. Different letters represent significant differences by one‐way ANOVA with Duncan's multiple range test (*P* < 0.05).

Given that both SlVQ15 and SlWRKY30IIc modulate tomato resistance to *M. incognita*, we wondered whether SlVQ15 and SlWRKY30IIc function synergistically. Accordingly, we crossed *slvq15‐c‐1* with *slwrky30IIc‐c‐2* to generate *slvq15 slwrky30IIc* double mutants. We inoculated the CM wild type, *slvq15‐c‐1*, *slwrky30IIc‐c‐2*, and *slvq15‐c‐1 slwrky30IIc‐c‐2* with *M. incognita*, and found that *slvq15‐c‐1 slwrky30IIc‐c‐2* double mutants were more sensitive to *M. incognita* than *slvq15‐c‐1* and *slwrky30IIc‐c‐2* single mutants, as indicated by the greater gall numbers per gram of roots and higher percentage of females than single mutants (Figure 4a,b).

*Proteinase Inhibitor II* (*PI‐II*) and *Plant Defence Factor* (*PDF*) are two RKN defence‐related genes (Xu *et al*., 2019). Without *M. incognita* infection, *SlPI‐II* and *SlPDF* expression levels were similar in the roots of CM wild type, *slvq15‐c‐1*, *slwrky30IIc‐c‐2*, and *slvq15‐c‐1 slwrky30IIc‐c‐2* (Figure 4c,d). *M. incognita* infection induced the *SlPI‐II* and *SlPDF* expressions in the CM wild type. Under *M. incognita* infection, *SlPI‐II* and *SlPDF* expression levels were lower in the roots of *slvq15‐c‐1* and *slwrky30IIc‐c‐2* single mutants than those in roots of CM wild type, whereas their expression was the lowest in the *slvq15‐c‐1 slwrky30IIc‐c‐2* double mutants (Figure 4c,d). The *slvq15 slwrky30IIc* double mutation severely disrupted *M. incognita* infection‐induced *SlPI‐II* and *SlPDF* expressions. These findings in Figure 4 indicated that SlVQ15 coordinately acts with SlWRKY30IIc to mediate defence against *M. incognita*.

Additionally, the *slvq15‐c‐1 slwrky30IIc‐c‐2* double mutants displayed similar phenotypes to the CM wild type in root development, plant growth, plant biomass, percentage of fruit set, and average weight per mature fruit (Figure S8), indicating that the loss of function of both SlVQ15 and SlWRKY30IIc may have no obvious impact on root development, plant growth, and fertility.

### SlJAZs associate with SlWRKY30IIc and affect the SlVQ15‐SlWRKY30IIc interaction

**Figure 5:**
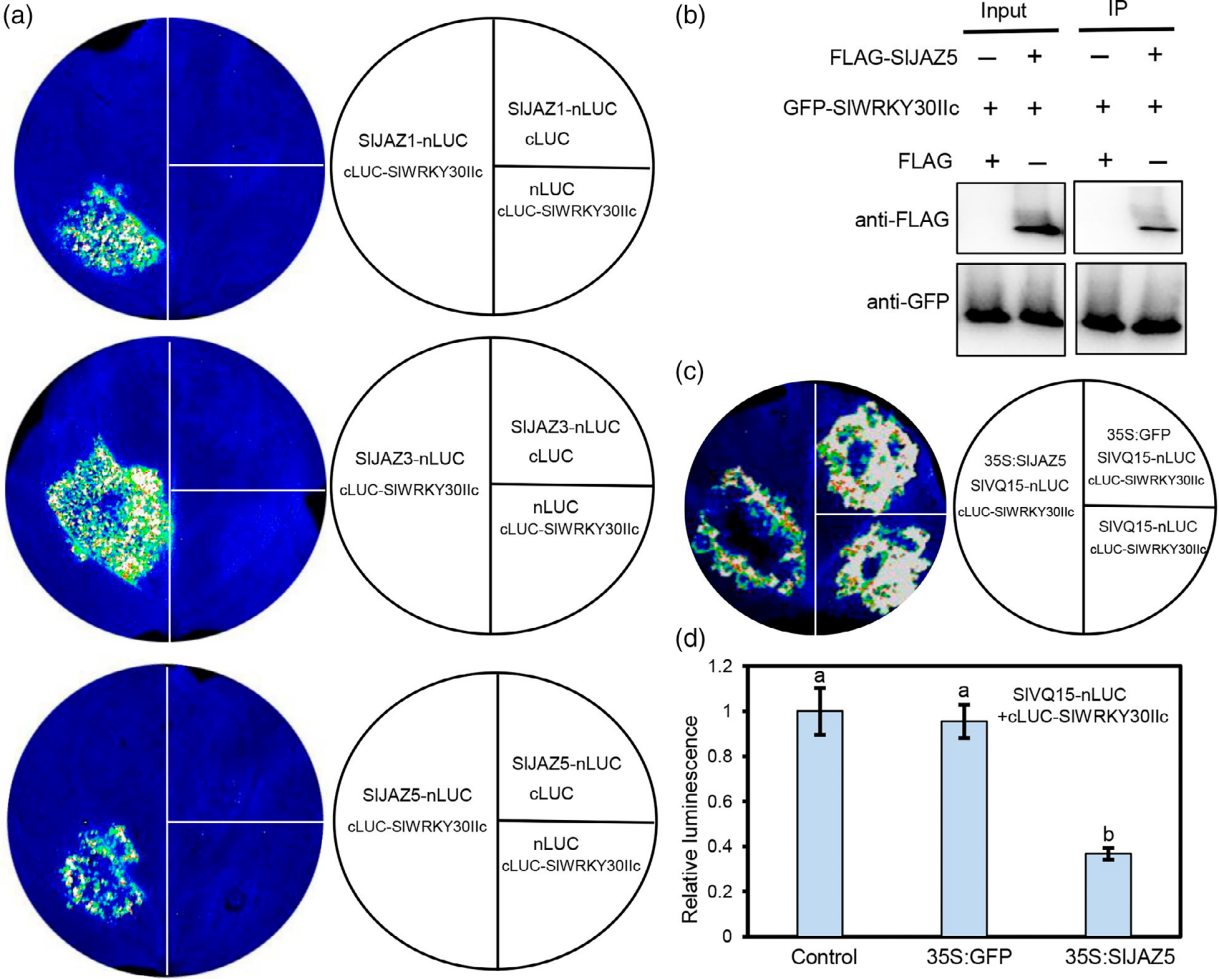
SlJAZs associate with SlWRKY30IIc and interfere with the SlWRKY30IIc‐SlVQ15 interaction. (a) LCI assays showing that SlWRKY30IIc interacts with SlJAZs. LUC activities were observed when the nLUC‐fused SlJAZs (SlJAZ1/SlJAZ3/SlJAZ5‐nLUC) and cLUC‐fused SlWRKY30IIc were coexpressed in *Nicotiana benthamiana* leaves. (b) Coimmunoprecipitation (Co‐IP) assay to confirm the interaction between SlWRKY30IIc and SlJAZ5. GFP‐fused SlWRKY30IIc was transiently coexpressed with FLAG‐empty (FLAG), FLAG‐fused SlJAZ5 (FLAG‐SlJAZ5) in *N. benthamiana* leaves for immunoprecipitation with anti‐GFP antibody‐conjugated agarose. Immunoblot analysis was carried out with anti‐FLAG and anti‐GFP antibodies. (c) The interaction of SlVQ15 with SlWRKY30IIc was inhibited by SlJAZ5. The indicated combinations SlVQ15‐nLUC/cLUC‐SlWRKY30IIc, SlVQ15‐nLUC/cLUC‐SlWRKY30IIc/35S:GFP, or SlVQ15‐nLUC/cLUC‐SlWRKY30IIc/35S:SlJAZ5 (with identical amounts of SlVQ15‐nLUC/cLUC‐SlWRKY30IIc) were infiltrated into *N. benthamiana* leaves. (d) Relative luminescence intensity in (c). Luminescence intensity was calculated using the values of 10 independent luminescence measurements. Values represent the means (±SE). Different letters represent significant differences by one‐way ANOVA with Duncan's multiple range test (*P* < 0.05).

Our previous study verified that SlVQ15 interacts with SlJAZs (Huang *et al*., 2022b). We wondered whether SlWRKY30IIc also associates with SlJAZs. LCI assays showed that coinfiltration of nLUC‐fused SlJAZ1, SlJAZ3, or SlJAZ5 (SlJAZ1‐nLUC, SlJAZ3‐nLUC, or SlJAZ5‐nLUC) and cLUC‐fused SlWRKY30IIc (cLUC‐SlWRKY30IIc) in *N. benthamiana* leaves resulted in LUC activity, while the negative controls did not (Figure 5a). Among these three SlWRKY30IIc interaction SlJAZs, only SlJAZ5 also associated with SlVQ15 (Figure 5a) (Huang *et al*., 2022b). Therefore, we selected SlJAZ5 as a representative for further study. We performed a coimmunoprecipitation (Co‐IP) assay to verify the interaction of SlJAZ5 and SlWRKY30IIc. GFP‐fused SlWRKY30IIc (GFP‐SlWRKY30IIc) was coexpressed with FLAG‐fused SlJAZ5 (FLAG‐SlJAZ5) or FLAG‐empty (FLAG) in *N. benthamiana* leaves for coimmunoprecipitation. The results in Figure 5b showed that FLAG‐SlJAZ5 was coimmunoprecipitated with GFP‐SlWRKY30IIc. The LCI and Co‐IP assays consistently suggested that SlJAZs interact with SlWRKY30IIc (Figure 5a,b).

In addition, we explored whether SlJAZs affect the protein stability of SlWRKY30IIc. Cell‐free degradation assay showed that the stabilities of transiently expressed myc‐SlWRKY30IIc proteins were similar when incubated with crude proteins from *N. benthamiana* leaves without or with FLAG‐fused SlJAZ5 expression (Figure S9a). Consistently, *in vivo* degradation assays demonstrated that SlJAZ5 had no effect on SlWRKY30IIc stability, and that the degradation rate of myc‐SlWRKY30IIc did not differ between the protoplasts without or with FLAG‐SlJAZ5 transfection (Figure S9b). These results suggested that SlJAZ5 could not alter SlWRKY30IIc stability.

We further conducted LCI assays to investigate the effects of SlJAZs on the SlVQ15‐SlWRKY30IIc interaction. When SlJAZ5 was coexpressed with SlVQ15‐nLUC/cLUC‐SlWRKY30IIc, LUC activity was clearly decreased compared with that only SlVQ15‐nLUC/cLUC‐SlWRKY30IIc were coexpressed (Figure 5c,d). As a negative control, coexpression of GFP with SlVQ15‐nLUC/cLUC‐SlWRKY30IIc did not affect LUC activity (Figure 5c,d). These results suggested that SlJAZs interfere with the SlVQ15‐SlWRKY30IIc association. Additionally, we investigated whether JA regulates the expression levels of *SlWRKY30IIc* and *SlVQ15*, and found that the transcript levels of *SlWRKY30IIc* and *SlVQ15* in the CM roots were largely induced by JA treatment (Figure S10).

### SlWRKY30IIc directly binds to the promoters of *SlJAZs* and represses their expression

We explored the effects of the SlVQ15‐SlWRKY30IIc module on the JA signal transduction pathway. We conducted qRT‐PCR to assess whether the module affects the expression of essential genes (e.g. *SlJAZs* and *SlMYC2*) in the JA signalling pathway. Under normal conditions, the expression of *SlJAZs* and *SlMYC2* in *slvq15 slwrky30IIc* double mutants was similar to that in CM wild type (Figures S11, S12). Notably, with *M. incognita* infection, the expression levels of *SlJAZ1*, *SlJAZ3*, *SlJAZ7*, *SlJAZ9*, *SlJAZ10*, and *SlJAZ11* were higher in the roots of *slvq15 slwrky30IIc* double mutants than in CM wild type (Figure S11), while the expression of remaining *SlJAZs* (*SlJAZ2*, *SlJAZ4*, *SlJAZ5*, *SlJAZ6*, *SlJAZ8*, and *SlJAZ12*) and *SlMYC2* was not influenced (Figures S11, S12). These results suggested that the SlVQ15‐SlWRKY30IIc module regulates the expression levels of *SlJAZs* under *M. incognita* infection.

**Figure 6:**
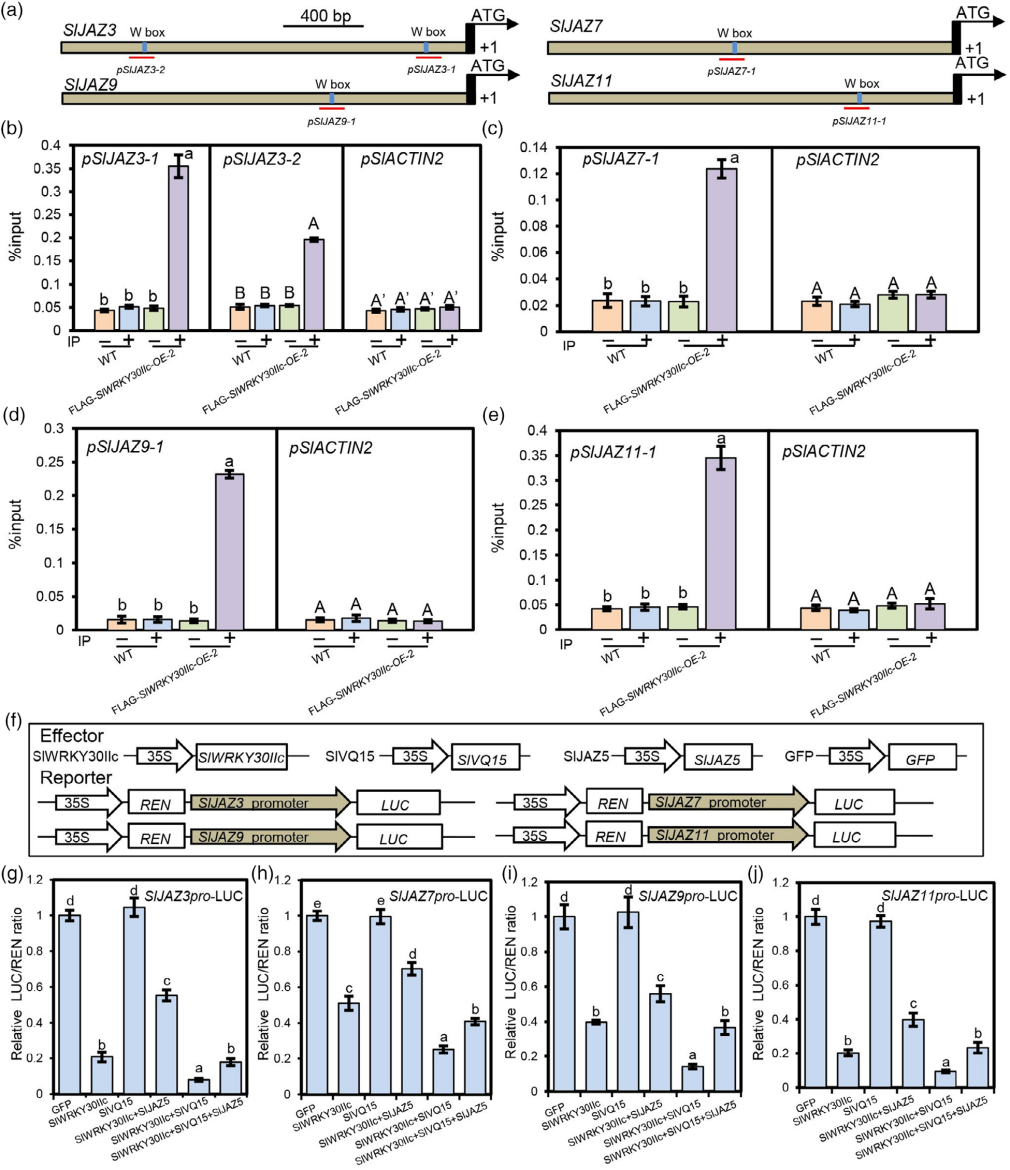
SlVQ15 enhances the transcriptional repression function of SlWRKY30IIc on *SlJAZs*, whereas SlJAZ5 inhibits this effect. (a) Diagram of the promoters of *SlJAZ3*, *SlJAZ7*, *SlJAZ9*, and *SlJAZ11*. The blue boxes represent the W‐box motif. The red lines indicate the regions of the *SlJAZs* promoters analysed in ChIP‐qPCR assays. (b–e) ChIP‐qPCR results showing that SlWRKY30IIc directly binds to the promoters of *SlJAZ3* (b), *SlJAZ7* (c), *SlJAZ9* (d), and *SlJAZ11* (e). The CM wild type and *SlWRKY30IIc*‐overexpressing plants (FLAG‐*SlWRKY30IIc‐OE‐2*) were inoculated with *M. incognita* for 24 h, and then the roots were, respectively, harvested for ChIP with goat anti‐mouse IgG (−) or anti‐flag antibody (+). qRT‐PCR was performed to detect the enrichment of the promoter regions in (a). The negative control was the *SlACTIN2* promoter. Data represent the means (±SE) of three independent biological replicates. Significant differences were analysed by one‐way ANOVA with Duncan's multiple range test (*P* < 0.05). (f) Schematic diagram of the constructs used in (g–j). LUC, firefly luciferase; REN, renilla luciferase. (g–j) SlWRKY30IIc attenuated the promoter activity of *SlJAZ3* (g), *SlJAZ7* (h), *SlJAZ9* (i), and *SlJAZ11* (j). Error bars represent the (±SE) of six independent biological replicates. Significant differences were analysed by one‐way ANOVA with Duncan's multiple range test (*P* < 0.05).

VQs cannot directly bind to DNA (Jing and Lin, 2015). Accordingly, we focused on whether SlWRKY30IIc directly binds to *SlJAZ1*, *SlJAZ3*, *SlJAZ7*, *SlJAZ9*, *SlJAZ10*, and *SlJAZ11*, and whether SlVQ15 affects SlWRKY30IIc function. By analysing the promoters of these six SlJAZs via PlantCARE, we found that the promoters of *SlJAZ3*, *SlJAZ7*, *SlJAZ9*, and *SlJAZ11* contain the W‐box motif (TTGACC/T) (Figure 6a), which is the typical WRKY TF binding sequence (Rushton *et al*., 2010). Chromatin immunoprecipitation quantitative PCR (ChIP‐qPCR) analysis revealed that the regions spanning the W‐box motifs of promoters of *SlJAZ3*, *SlJAZ7*, *SlJAZ9*, and *SlJAZ11* were enriched in FLAG*‐SlWRKY30IIc*‐*OE* plants under *M. incognita* infection (Figure 6b–e), suggesting that SlWRKY30IIc specifically binds to the W‐box motif of *SlJAZ3*, *SlJAZ7*, *SlJAZ9*, and *SlJAZ11* promoters.

### SlVQ15 promotes the transcriptional activity of SlWRKY30IIc, while SlJAZ5 attenuates this effect

We carried out Dual‐LUC assays using protoplasts, which was isolated from leaves of *M. incognita*‐infected tomato, to assess the regulatory effects of SlWRKY30IIc and SlVQ15‐SlWRKY30IIc on the individual promoters of *SlJAZ3*, *SlJAZ7*, *SlJAZ9*, and *SlJAZ11* (Figure 6f–j). Compared with GFP, the SlWRKY30IIc expression decreased the relative LUC/Renilla luciferase (REN) ratio in which *LUC* was driven by the respective promoter of *SlJAZ3*/*7*/*9/11*, implying that SlWRKY30IIc represses *SlJAZ3*, *SlJAZ7*, *SlJAZ9*, and *SlJAZ11* under *M. incognita* infection.

On the other hand, SlVQ15 expression alone did not affect the promoter activity of *SlJAZ3*/*7*/*9/11* in contrast to GFP, while SlVQ15 enhanced the inhibitory effect of SlWRKY30IIc on *SlJAZ3*/*7*/*9*/*11*, as SlVQ15/SlWRKY30IIc coexpression resulted in further decreased LUC/REN ratio compared with SlWRKY30IIc alone (Figure 6f–j), consistently supporting that SlVQ15 alone cannot bind to promoters to affect their activity and that SlVQ15 functions through SlWRKY30IIc.

We selected SlJAZ5 as a representative to analyse how SlJAZs affect the transcriptional activity of SlWRKY30IIc and SlVQ15/SlWRKY30IIc on *SlJAZ* promoters (Figure 6f–j). The results showed that SlJAZ5 interfered with the repressive effect of SlWRKY30IIc and SlVQ15/SlWRKY30IIc on the promoters of *SlJAZ3/7/9*/*11*, leading to derepression of promoter activity of *SlJAZ3*/*7*/*9*/*11*.

Taken together, these findings (Figure 6) demonstrated that SlWRKY30IIc binds to the promoters of *SlJAZs* and represses their expression, and that SlVQ15 enhances the transcriptional repression activity of SlWRKY30IIc, whereas SlJAZ5 attenuates these inhibitory effects.

### SlMYC2 binds to the promoter of *SlWRKY30IIc* and represses its expression

The SlJAZ‐interacting protein SlMYC2 is a negative regulator of resistance to RKNs (Du *et al*., 2017; Xu *et al*., 2019). ChIP‐seq data indicated that SlWRKY30IIc, but not SlVQ15, may be a SlMYC2‐targeted gene (Du *et al*., 2017).

**Figure 7:**
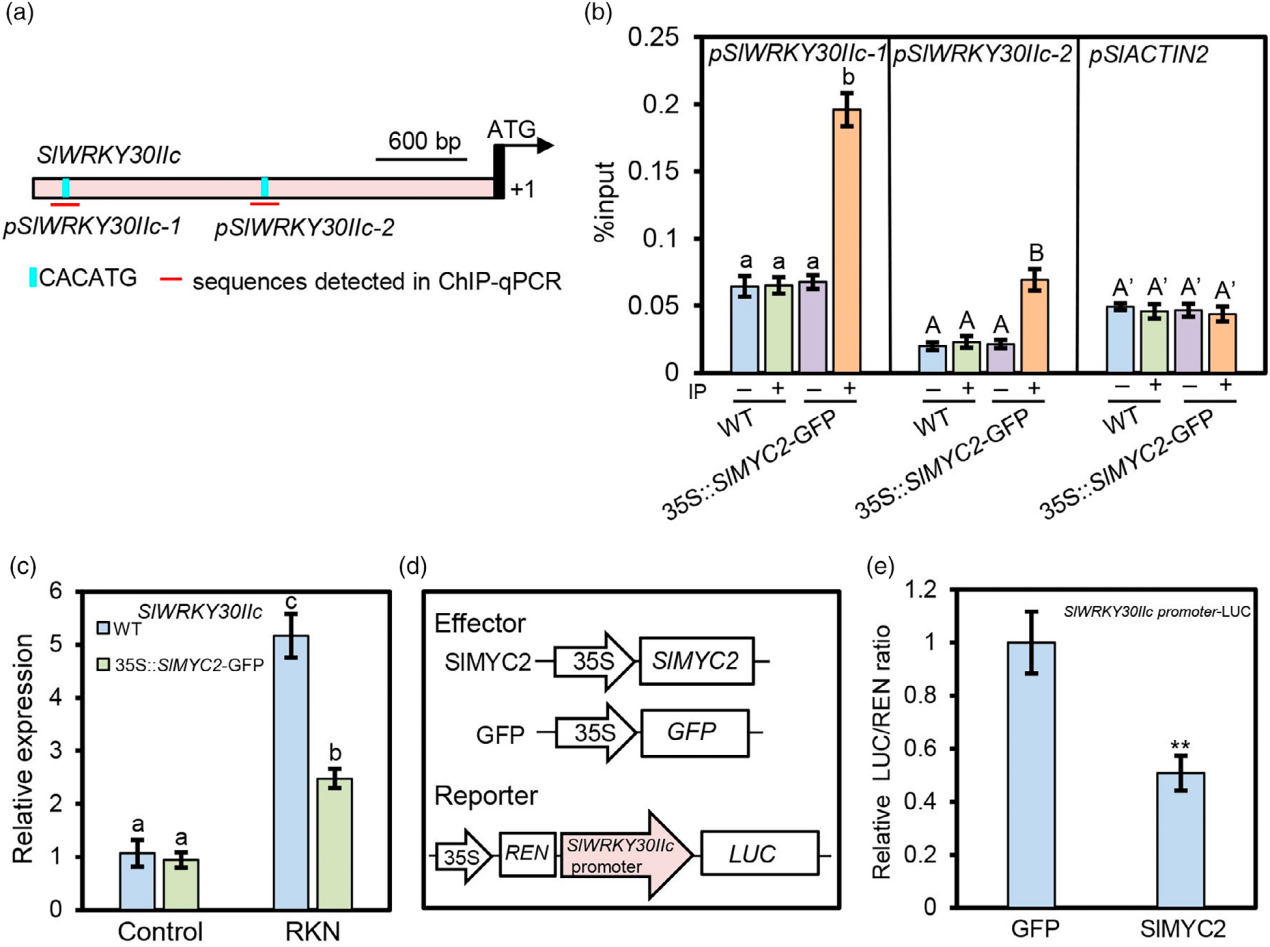
SlMYC2 binds to the *SlWRKY30IIc* promoter and inhibits its expression. (a) Diagram of *SlWRKY30IIc* promoter. (b) ChIP‐qPCR results showing that SlMYC2 directly binds to the *SlWRKY30IIc* promoter. M82 wild type and 35S::*SlMYC2*‐GFP plants were inoculated with *M. incognita* for 24 h, and the roots were harvested for ChIP with goat anti‐mouse IgG (−) or anti‐GFP antibody (+). qRT‐PCR was performed to detect the enrichment of the promoter regions indicated in (a). The negative control was the *SlACTIN2* promoter. Data represent the means (±SE) of three independent biological replicates. Significant differences were analysed by one‐way ANOVA with Duncan's multiple range test (*P* < 0.05). (c) Relative expression level of *SlWRKY30IIc* in roots of M82 wild type and 35S::*SlMYC2*‐GFP plants without (control) or with *M. incognita* infection for 24 h. Data represent the means (±SE) of three independent biological replicates. Letters represent significant differences by one‐way ANOVA with Duncan's multiple range test (*P* < 0.05). (d) Schematic diagram of the constructs used in (e). LUC, firefly luciferase; REN, renilla luciferase. (e) SlMYC2 repressed the *SlWRKY30IIc* promoter. Error bars represent the (±SE) of six independent biological replicates. Significant differences were analysed by Student's *t*‐test (**P < 0.01).

Two SlMYC2‐binding sequences (CACATG) are located within 3000 bp upstream of the start codon of *SlWRKY30IIc* (Figure 7a) (Du *et al*., 2017). To examine whether SlMYC2 can bind to the *SlWRKY30IIc* promoter via these two motifs, we used the roots of *M. incognita*‐infected M82 wild type and 35S::*SlMYC2*‐GFP to perform ChIP‐qPCR analysis. The results in Figure 7b demonstrated that SlMYC2 is bound to the CACATG motifs in *SlWRKY30IIc* promoter. Consistently, under *M. incognita* inoculation, the expression level of *SlWRKY30IIc* in the roots of 35S::*SlMYC2*‐GFP plants was significantly lower than that in M82 wild type, demonstrating that *SlMYC2* overexpression inhibits *M. incognita* infection‐induced *SlWRKY30IIc* expression (Figure 7c). In support of this, Dual‐LUC assays using protoplasts isolated from *M. incognita*‐infected tomato showed that SlMYC2 inhibited *SlWRKY30IIc* promoter activity (Figure 7d,e).

We also wondered whether SlMYC2 could also bind to the promoter of *SlVQ15*. Our ChIP‐qPCR analysis in Figure S13 indicated that *SlVQ15* is not a target gene of SlMYC2. Taken together, these data suggest that SlMYC2 binds to the promoter and represses the expression of *SlWRKY30IIc*, but not those of *SlVQ15*.

## Discussion

The ruinous agricultural pest RKNs severely damage crop yields. However, green and efficient preventative strategies for RKNs are lacking. Exploring the molecular mechanism underlying RKNs tolerance of the host will provide a sustainable and feasible opportunity to improve the ability of defending against RKNs.

**Figure 8:**
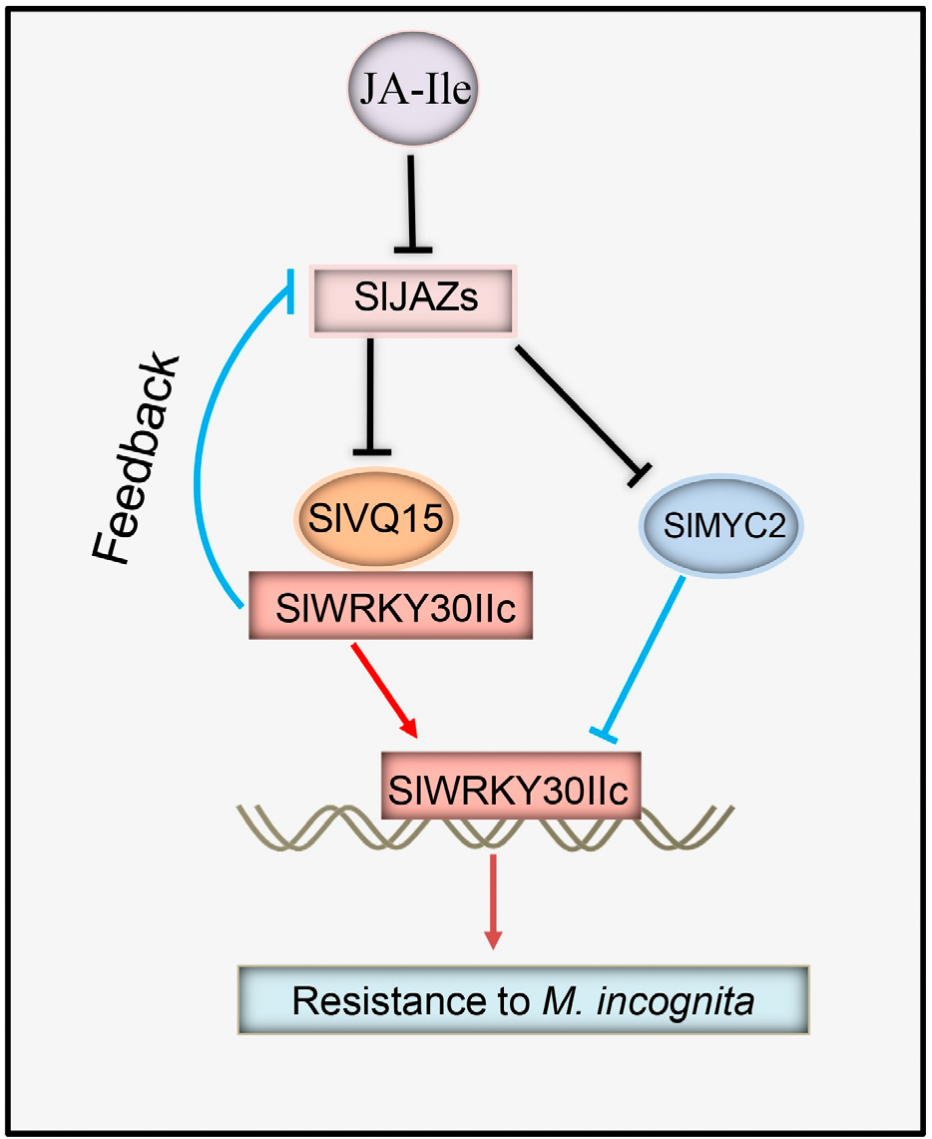
A simplified model of the SlVQ15‐SlWRKY30IIc module in resistance to the RKN *M. incognita* in tomato. SlVQ15 recruited and stabilized SlWRKY30IIc. They synergistically controlled tomato resistance to the RKN *M. incognita*. The interaction of SlVQ15 and SlWRKY30IIc was interfered by the JA signalling repressors SlJAZs. In turn, SlWRKY30IIc bound to the promoters of *SlJAZs* and inhibited their expression, which was promoted by SlVQ15 and attenuated by SlJAZs. Thereby, it formed a feedback loop to fine‐tune tomato resistance to *M. incognita*. Additionally, the expression of *SlWRKY30IIc* was repressed by the SlJAZ‐interacting protein SlMYC2, a negative controller in defending against *M. incognita*.

Our findings revealed new insights on mechanism of the SlVQ15‐SlWRKY30IIc module in tomato defence against the RKN *M. incognita* (Figure 8). SlVQ15 recruited and stabilized SlWRKY30IIc, and acted as an activation partner of SlWRKY30IIc. They positively and coordinately modulated resistance to the RKN *M. incognita* in tomato without influencing plant growth, development, and fertility. The SlVQ15 and SlWRKY30IIc interaction was interfered by the JA signalling repressors SlJAZs, while SlWRKY30IIc directly inhibited *SlJAZs* expression. Additionally, the transcript level of *SlWRKY30IIc* was attenuated by the negative controller SlMYC2 in defending against *M. incognita*. It provided candidate genes, genetic resources, and guidance for the control of RKNs, which will benefit the genetic improvement and breeding of tomato and other crops for resistance to RKNs.

VQ proteins link with different components to achieve their distinct biological functions. For instance, AtVQ23 and AtVQ16 interact with AtWRKY75 to regulate seed germination and leaf senescence, and associate with AtWRKY33 and AtWRKY57 to fine‐tune resistance to *B. cinerea* (Jiang and Yu, 2016; Lai *et al*., 2011; Zhang *et al*., 2022). AtVQ29 interacts with PIF1 to repress seedling deisolation and forms heterodimers with AtVQ12 to negatively modulate defence against *B. cinerea* (Li *et al*., 2014; Wang *et al*., 2015). SlVQ15 associates with SlWRKY31 to regulate resistance to *B. cinerea* and recruits SlWRKY30IIc to control defence against RKNs (Huang *et al*., 2022b) (Figures 1, 2, 3, 4). Isolation of novel SlVQ15 partners is of great significance for fully understanding its biological functions.

The agricultural production of other Solanaceae plants, such as potato (*Solanum tuberosum*) and pepper (*Capsicum annuum*), is also destructed by RKNs (Hu *et al*., 2020; Lima *et al*., 2018). SlVQ15 shares 75.8% and 65.7% identity with StVQ22 and CaVQ22, and SlWRKY30IIc is also highly conserved with 95.4% and 81.2% identity to StWRKY71 and CaWRKY71, respectively (Figure S14), suggesting that they probably function in a conserved manner in defence against RKNs in potato and pepper. It would be significant to investigate whether the homologues of SlVQ15 and SlWRKY30IIc in other species participate in defence against RKNs, which will largely broaden their potential agricultural applications. Interestingly, our Y2H screening showed that SlVQ22 was a candidate SlVQ15 interaction protein (Table S1). It will be interesting to investigate whether SlVQ22 forms heterodimers with SlVQ15 or assembles a ternary complex with SlWRKY30IIc/SlVQ15 and regulates defence against RKNs.

SlWRKY30IIc was also named SlWRKY16 (Kumar *et al*., 2023). *Rhizobium rhizogenes*‐mediated transient *SlWRKY16* overexpression in hairy roots reduced defence against the RKN *M. javanica* (Kumar *et al*., 2023), whereas we showed that SlWRKY30IIc enhanced resistance to the RKN *M. incognita* by using stable transgenic overexpression lines and gene‐editing mutants (Figure 3). These seemingly discrepant results might be caused by interspecies genetic differences of RKNs or tomato genetic backgrounds (Khan *et al*., 2023; Rutter *et al*., 2022), suggesting the complexity of plant–nematode interactions and emphasizing the need for future research on the underlying mechanisms of host response to distinct RKNs.

Some regulators have been identified as interaction partners of SlJAZs to control JA‐mediated tomato defence against *M. incognita* (Du *et al*., 2017; Huang *et al*., 2022b; Shang *et al*., 2019; Xu *et al*., 2019). Unlike these controllers, the SlVQ15‐SlWRKY30IIc module not only acts as a SlJAZ‐interacting complex but also regulates *SlJAZs* expression, forming a feedback loop to fine‐tune RKNs resistance (Figures 1, 2, 3, 4, 5, 6, 7). It would be interesting to investigate whether there are other SlJAZ partners that contribute to tomato defence against *M. incognita*. Additionally, it is worth investigation of the relationship between the SlVQ15‐SlWRKY30IIc module and other SlJAZ‐interacting proteins (e.g. whether they control the same target genes).

The relationships of MYC2 and WRKYs have been investigated in several previous studies. AtMYC2 and AtWRKY20 both interact with begomoviral βC1 protein, whereas they directly bind to the promoters of each other to mutually inhibit their expression, forming a regulatory loop of the parasite–vector–host tripartite interactions (Zhao *et al*., 2019). AtMYC2 interacts with AtWRKY46, and they play vital roles in volatile organic compound‐induced defence responses (Hao *et al*., 2024). Here, we found that SlMYC2 bound to the *SlWRKY30IIc* promoter, and inhibited *M. incognita* infection‐induced *SlWRKY30IIc* (Figure 7b–e). Upon RKNs infection, tomato plants induce *SlWRKY30IIc* expression to enhance defence, whereas SlMYC2 was upregulated to inhibit this effect (Figures 3, 4, 7, Figure S4b) (Kumar *et al*., 2023; Xu *et al*., 2019), implying that dynamic coordinated expression of positive and negative regulators may minimize excessive defence and lower resource cost for appropriate adaptation to the fluctuating RKN infection pressure, or that RKNs somehow hijack *SlMYC2* expression to inhibit host defence and favour its infection, which are interesting to be investigated. Additionally, whether the SlVQ15‐SlWRKY30IIc module physically interact with or directly bind to the promoter of SlMYC2 as a layer of regulation remains to be elucidated.

The so‐far discovered tomato genes regulating defence against *M. incognita* can be categorized into three types according to their effects on growth. Some have unfavourable impacts on plant growth/development (e.g. SlMYB108, SlMYB112, and SlFLS) (Zhao *et al*., 2023), and others have unclear effect on growth/development (e.g. SlERF1 and SlCSN4/5) (Shang *et al*., 2019; Zou *et al*., 2023). The remaining, including SlVQ15/SlWRKY30IIc, SlHsfA1a, and SlRING1, exhibit no growth penalty and have application potential for tomato breeding in terms of defence against RKNs (Figures 1, 3, 4, Figures S2, S7, S8) (Zhou *et al*., 2018; Zou *et al*., 2022). Using these RKNs‐resistant factors as clues to screen interacting proteins or target genes will obtain genes specifically for resistance to RKNs. The identified genes and mechanisms would provide valuable information on molecular breeding for the control of RKNs in tomato and other crops. Future research will facilitate improvements in RKN stress tolerance of crops through genetic manipulation, thereby paving the way for green and safe methods for RKN control.

## Materials and methods

### Plant materials and growth conditions

The wild‐type tomato (*Solanum lycopersicum*) used in this study was Castlemart (CM) except for 35S::*SlMYC2*‐GFP. For 35S::*SlMYC2*‐GFP, cv M82 was used as the wild type. The *SlVQ15OE* lines, *slvq15* mutants, and 35S::*SlMYC2*‐GFP were described previously (Du *et al*., 2017; Huang *et al*., 2022b).

To generate FLAG‐*SlWRKY30IIc‐OE* transgenic plants, the coding sequence of *SlWRKY30IIc* was cloned into a modified pCAMBIA1300 vector containing three FLAG tags with the control of the *35S* promoter. The resulting vector was transferred into *Agrobacterium tumefaciens* GV3101 and then transformed into CM plants. Transformants were selected according to their resistance to hygromycin B. FLAG‐*SlWRKY30IIc‐OE* T3 homozygotes were used for further study.

To generate *slwrky30IIc* mutants, two guide RNA target sites in the first and second exons of *SlWRKY30IIc* were chosen for genome editing. The target sequences were inserted into the pCBSG012 vector via the *Bsa* I site as previously described (Huang *et al*., 2022a). The construct was subsequently transformed into CM plants by *Agrobacterium*‐mediated transformation. Cas9‐free *slwrky30IIc* mutants without off‐target effects were identified by PCR, confirmed by DNA sequencing, and used in this study. The primer pairs used for vector construction and identification of *slwrky30IIc* mutants are listed in Tables S2–S5. The *slvq15‐c‐1 slwrky30IIc‐c‐2* double mutants were generated by genetic crossing with *slvq15‐c‐1* and *slwrky30IIc‐c‐2*.

Tomato plants were grown at 24–26 °C/16–18 °C (day/night) with a 16 h photoperiod. *Nicotiana benthamiana* was grown at 25–28 °C/16–18 °C (day/night) with a 16 h photoperiod.

### Quantitative real‐time PCR

For Figures 1a, 4c,d, 7c, S1, S4b, S11, and S12, total RNA was isolated from the roots of the indicated plants without or with inoculation of *M. incognita*. For Figure S4c, total RNA was extracted from roots, stems, leaves, flowers, and fruits of CM wild type. For Figure S5, total RNA was isolated from the leaves of CM wild type and *SlWRKY30IIc*‐overexpressing plants. For Figure S10, total RNA was isolated from the roots of CM wild type with 100 μm MeJA treatment. RNA extraction was performed using an RNA Extraction Kit (Tiangen, Beijing, China) according to the manufacturer's instructions, and cDNA was synthesized with the kits (Transgen, Beijing, China) following the manufacturer's instructions. The qRT‐PCR experiments were carried out on a Bio‐Rad CFX96 qPCR instrument using SYBR Green Mix (TaKaRa, Kusatsu, Japan). Tomato *ACTIN2* was used as an internal control, and the relative expression level was calculated using the previously described 2^−ΔΔCT^ method (Livak and Schmittgen, 2001). Primers for qRT‐PCR are listed in Table S6. The experiments were conducted in three biological replicates.

### Subcellular localization

The full‐length coding region of SlWRKY30IIc was inserted into the pEGAD vector to generate the GFP‐SlWRKY30IIc vector. The resulting vector was injected into *N. benthamiana* leaves through *Agrobacterium* (GV3101)‐mediated transformation. Then 10 μg/mL 4′,6‐diamidino‐2‐phenylindole (DAPI) was injected into tobacco leaves 2 h before observation. GFP and DAPI fluorescences were detected using a laser confocal microscope (TCS‐SP5; Leica, Wetzlar, Germany). The primers used for vector construction are listed in Table S2.

### Y2H screening and Y2H assays

For Y2H screening, 28‐day‐old CM seedlings were infected with approximately 400 *M. incognita* J2s for 24 h, and the roots were harvested for mRNA isolation and cDNA library construction (Oebiotech, Shanghai, China). The coding sequence of SlVQ15 was cloned into the pLexA vector containing the DNA binding domain (BD) as bait. Y2H screening was performed according to the manufacturer's instructions (Clontech, Mountain View, CA, USA). After screening, 325 interactions were tested, and among these interactions, 68 positive colonies were isolated. Plasmids from these positive colonies were transformed into *E. coli* for identification and sequencing. The information for each candidate is listed in Table S1.

For Y2H assays, the full‐length coding sequences of SlVQ15 and its related variants were inserted into the pLexA vector with the BD domain. The conserved VQ residues in the SlVQ15‐NT2^Mu‐VQ motif^ variant were mutated to AA residues using Fast MultiSite Mutagenesis System (Transgene, Beijing, China). The full‐length coding sequence of SlWRKY30IIc and its related domains were individually inserted into the pB42AD vector, which contains the activation domain (AD). The yeast strain EGY48 was cotransformed with the corresponding BD and AD vectors, and subsequently the transformed yeast cells were grown on 2% Gal/1% raffinose/SD/−Ura/−His/−Trp/−Leu/X‐β‐Gal plates for 3 days at 30 °C to analyse protein interactions. Primers used for vector constructs are listed in Table S2. The experiments were conducted in three biological replicates.

### LCI assays

The full‐length coding sequences of SlVQ15, SlVQ15‐NT, SlJAZ1, SlJAZ3, and SlJAZ5 were inserted into the pCAMBIA‐nLUC vector (with a modification to have a start codon) to produce SlVQ15‐nLUC, SlVQ15‐NT‐nLUC, SlJAZ1‐nLUC, SlJAZ3‐nLUC, and SlJAZ5‐nLUC. The full‐length coding sequences of SlWRKY30IIc, and SlWRKY30IIc‐CT, were cloned into the pCAMBIA‐cLUC vector to generate cLUC‐SlWRKY30IIc and cLUC‐SlWRKY30IIc‐CT. *Agrobacterium tumefaciens* strains (GV3101) with the corresponding nLUC and cLUC‐fused protein pairs were injected into the leaves of *N. benthamiana*. Fifty hours later, the injected tobacco leaves were sprayed with a luciferin solution (0.1 mm luciferin, 0.1% Tween 20, 1 mm NaOH), and luciferase (LUC) luminescence was captured by a Tanon 5200Multi instrument (Tanon, Shanghai, China). Primers used for vector constructs are listed in Table S2. The experiments were conducted in three biological replicates.

### Pull‐down assays

MBP‐fused SlVQ15 was purified as previously described (Huang *et al*., 2022b). FLAG‐fused SlWRKY30IIc protein was transiently expressed in *N. benthamiana* leaves and extracted as previously described (Huang *et al*., 2022b). For pull‐down assays, the amylose resin was incubated with MBP‐fused SlVQ15 and the negative control MBP proteins at 4 °C for 2 h. Then, FLAG‐fused SlWRKY30IIc protein was added and incubated at 4 °C for 2 h. After washing, the samples were resuspended in SDS loading buffer, boiled at 95 °C for 5 min, and then assessed by immunoblotting using an anti‐FLAG antibody (Abmart, Shanghai, China). Primers used for vector constructs are listed in Table S2. The experiments were conducted in three biological replicates.

### Co‐IP assays

The CDS of SlJAZ5 was cloned into a modified pCAMBIA1300 vector to construct a FLAG‐fused SlJAZ5 vector. The indicated *Agrobacterium tumefaciens* pairs (GFP‐SlWRKY30IIc with FLAG‐empty or FLAG‐SlJAZ5) were injected into the leaves of *N. benthamiana*. Fifty hours later, the total protein was extracted with a buffer (50 mm Tris–HCl, pH 7.5, 2 mm DTT, 100 mm NaCl, 1 mm phenylmethylsulfonyl fluoride, 0.1% Tween 20, 50 mm MG132, and complete protease inhibitor cocktail), concentrated and incubated with anti‐GFP agarose beads (Abmart, Shanghai, China) for 2 h at 4 °C. After three washes, the samples were boiled with SDS loading buffer for analysis. Anti‐GFP (Abmart) and anti‐FLAG (Abmart) antibodies were used for immunoblotting.

### Protein degradation assays

SlWRKY30IIc was cloned into pROK2 vector to generate myc‐fused SlWRKY30IIc. For Figure 2h and Figure S9a, *Agrobacterium tumefaciens* carrying GFP‐SlWRKY30IIc or myc‐SlWRKY30IIc was injected into the leaves of *N. benthamiana*. Fifty hours after injection, the GFP‐SlWRKY30IIc and myc‐SlWRKY30IIc proteins were extracted, purified by anti‐GFP agarose beads or anti‐myc agarose beads (Abmart). Purified GFP‐SlWRKY30IIc was incubated with crude protein from CM wild type and *SlVQ15*‐overexpressing lines in the presence of 1 mm ATP for the indicated time at 25 °C. Purified myc‐SlWRKY30IIc was incubated with crude protein from *N. benthamiana* leaves injected without or with FLAG‐SlJAZ5 in the existence of 1 mm ATP for the indicated time at 25 °C.

For *in vivo* protein degradation assays in Figure 2i, myc‐SlWRKY30IIc and GFP were cotransformed into CM wild type (WT) and *slvq15* mutant (*slvq15‐c‐1*) protoplasts according to the previously described method (Yoo *et al*., 2007). For Figure S9b, myc‐SlWRKY30IIc plus GFP, or myc‐SlWRKY30IIc plus FLAG‐SlJAZ5 and GFP were cotransformed into CM wild‐type protoplasts. GFP was coexpressed to serve as an internal control. After incubated for 16 h, the protoplasts were treated with 50 μm CHX. Samples were collected at the indicated time points.

The samples were then separated on SDS‐PAGE gels and detected by immunoblotting with an anti‐GFP antibody, an anti‐myc antibody, an anti‐FLAG antibody (Abmart), or an anti‐β‐actin antibody (CWBIO, Jiangsu, China) as indicated.

### *Meloidogyne incognita* inoculation assays

*Meloidogyne incognita* eggs were collected from infected‐CM tomato roots using the needle of a syringe and incubated at 28 °C for 2–4 days to hatch J2s. Twenty‐eight‐day‐old tomato seedlings (CM wild type, T3 homozygous *SlVQ15OE* and *FLAG‐SlWRKY30IIc‐OE* plants, Cas9‐free T2 homozygous *slvq15* and *slwrky30IIc*, and *slvq15 slwrky30IIc* double mutants) were inoculated with approximately 400 J2s. At 14 days or 21 days after infection, the gall numbers were calculated as previously described (Huang *et al*., 2022a). To calculate the percentage of females, the 35‐days‐infected tomato roots were washed, frozen and thawed 4–5 times, and blended. Then, the sample is filtered through two‐layer sieves (on top is the sieve with 200 mesh per square inch, and below is the sieve with 600 mesh per square). The nematodes were placed in the mesh and collected. The numbers of J2s, J3/J4s, and females were counted under a microscope (SMZ‐140; Motic, Guangdong, China) in accordance with a previously described method (Li *et al*., 2017). The experiments were conducted in three biological replicates.

### ChIP assays

ChIP assays were conducted with the EpiQuik Plant ChIP kit (Epigentek, Farmingdale, NY, USA) according to the manufacturer's instructions. Briefly, 21‐day‐old of the indicated seedlings in Figures 6b–e, 7b, Figure S13 were inoculated with *M. incognita* for 24 h. The roots of the plants were crosslinked with 1% formaldehyde and then neutralized with 0.125 m glycine. The anti‐FLAG (Abmart) or anti‐GFP antibody (Abmart) was used to immunoprecipitate the chromatin protein. Goat anti‐mouse IgG (Abmart) was used as a negative control. qRT‐PCR analysis was carried out to assess the enriched promoter fragments by the previously described method (Yamaguchi et al., 2014). The *SlACTIN2* promoter was used as a negative control. Primers for qRT‐PCR in the experiment are listed in Table S6. The experiments were conducted in three biological replicates.

### Dual‐LUC assays

The promoters of *SlWRKY30IIc*, *SlJAZ3*, *SlJAZ7*, *SlJAZ9*, and *SlJAZ11* were inserted into the pGREENII 0800 LUC vector, and SlMYC2, SlWRKY30IIc, SlVQ15, SlJAZ5, and GFP were cloned into the pGreenII 62‐SK vector (Hellens *et al*., 2005). CM wild type was infected with *M. incognita* for 24 h, and then harvested for protoplast extraction and transfection as described previously (Yoo *et al*., 2007). Twenty hours after the corresponding pairs were transformed into protoplasts, the activities of firefly luciferase (LUC) and renilla luciferase (REN) were detected by a dual‐luciferase assay kit (Promega, Madison, WI, USA). Primers used for vector constructs are listed in Table S2.

### Analysis of root phenotypes

The roots of 40‐day‐old CM wild type and *slvq15‐c‐1 slwrky30IIc‐c‐2* double mutants were recorded by an Expression 12000XL Root Scanner (Epson, Nagano, Japan) and analysed using the WinRhizo software (Regent Instruments, Quebec, Canada).

### Accession numbers

The accession numbers for the genes are as follows: SlWRKY30IIc (Solyc07g056280), SlVQ15 (Solyc07g043250), SlJAZ1 (Solyc07g042170), SlJAZ2 (Solyc12g009220), SlJAZ3 (Solyc03g122190), SlJAZ4 (Solyc12g049400), SlJAZ5 (Solyc03g118540), SlJAZ6 (Solyc01g005440), SlJAZ7 (Solyc11g011030), SlJAZ8 (Solyc06g068930), SlJAZ9 (Solyc08g036640), SlJAZ10 (Soly08g036620), SlJAZ11 (Solyc08g036660), SlJAZ12 (Solyc01g009740), SlMYC2 (Solyc08g076930), and SlACTIN2 (Solyc11g005330).

## Conflict of interest

The authors declare no conflicts of interest.

## Author contributions

S.S., S.W., and H.H. conceived the project. S.S. and H.H. wrote the article. H.H., X.M., L.S., Y.W., J.M., Y.H., and M.Z. performed the research. H.H., X.M., W.Z., and R.Y. analysed the data.

## Supporting information


**Figure S1** Expression levels of *SlVQs* in response to the RKN *Meloidogyne incognita* infection.
**Figure S2** *SlVQ15* could not regulate tomato growth and fertility.
**Figure S3** The interaction of domains from SlWRKY30IIc and SlVQ15.
**Figure S4** Subcellular localization and expression pattern of SlWRKY30IIc.
**Figure S5** Quantitative real‐time PCR analysis of *SlWRKY30IIc* in the *SlWRKY30IIc*‐overexpressing plants.
**Figure S6** Generation of *slwrky30IIc* mutants using CRISPR/Cas9 technology.
**Figure S7** *SlWRKY30IIc* could not control tomato growth and fertility.
**Figure S8** The phenotypes of *slvq15 slwrky30IIc* double mutants.
**Figure S9** SlJAZ5 could not affect the protein stability of SlWRKY30IIc.
**Figure S10** JA induced the expression of *SlWRKY30IIc* and *SlVQ15*.
**Figure S11** *SlJAZs* expression in the CM wild type and *slvq15 slwrky30IIc* mutants with the RKN *M. incognita* infection.
**Figure S12** *SlMYC2* expression in the CM wild type and *slvq15 slwrky30IIc* mutants with the RKN *M. incognita* infection.
**Figure S13** SlMYC2 could not bind to the typical G‐box‐like motif in the *SlVQ15* promoter.
**Figure S14** Sequence alignment of SlVQ15 or SlWRKY30IIc and their respective homologues in *Solanum tuberosum* and *Capsicum annuum*.

**Table S1** Positive clones of SlVQ15 interaction candidates in Y2H screening assay.
**Table S2** Primers used for vector construction.
**Table S3** Primers used for analysis of the target site mutation.
**Table S4** Primers used for analysis of the existence of *Cas9*.
**Table S5** Primers used for analysis of off‐target site mutation.
**Table S6** Primers used for qRT‐PCR analysis.

## Data Availability

All the data generated in this study are available upon request.
