# Supplementary material for: SlVQ15 recruits SlWRKY30IIc to link with jasmonate pathway in regulating tomato defence against root‐knot nematodes
Source: Plant Biotechnol J. 2024 Nov 5;23(1):235–49. doi: 10.1111/pbi.14493 (PMC11672745; doi:10.1111/pbi.14493)
Supplement: Supplementary file 1 — Figure S1 Expression levels of SlVQs in response to the RKN Meloidogyne incognita infection. Figure S2 SlVQ15 could not regulate tomato growth and fertility. Figure S3 The interaction of domains from SlWRKY30IIc and SlVQ15. Figure S4 Subcellular localization and expression pattern of SlWRKY30IIc. Figure S5 Quantitative real‐time PCR analysis of SlWRKY30IIc in the SlWRKY30IIc‐overexpressing plants. Figure S6 Generation of slwrky30IIc mutants using CRISPR/Cas9 technology. Figure S7 SlWRKY30IIc could not control tomato growth and fertility. Figure S8 The phenotypes of slvq15 slwrky30IIc double mutants. Figure S9 SlJAZ5 could not affect the protein stability of SlWRKY30IIc. Figure S10 JA induced the expression of SlWRKY30IIc and SlVQ15. Figure S11 SlJAZs expression in the CM wild type and slvq15 slwrky30IIc mutants with the RKN M. incognita infection. Figure S12 SlMYC2 expression in the CM wild type and slvq15 slwrky30IIc mutants with the RKN M. incognita infection. Figure S13 SlMYC2 could not bind to the typical G‐box‐like motif in the SlVQ15 promoter. Figure S14 Sequence alignment of SlVQ15 or SlWRKY30IIc and their respective homologues in Solanum tuberosum and Capsicum annuum. [file PBI-23-235-s001.docx]

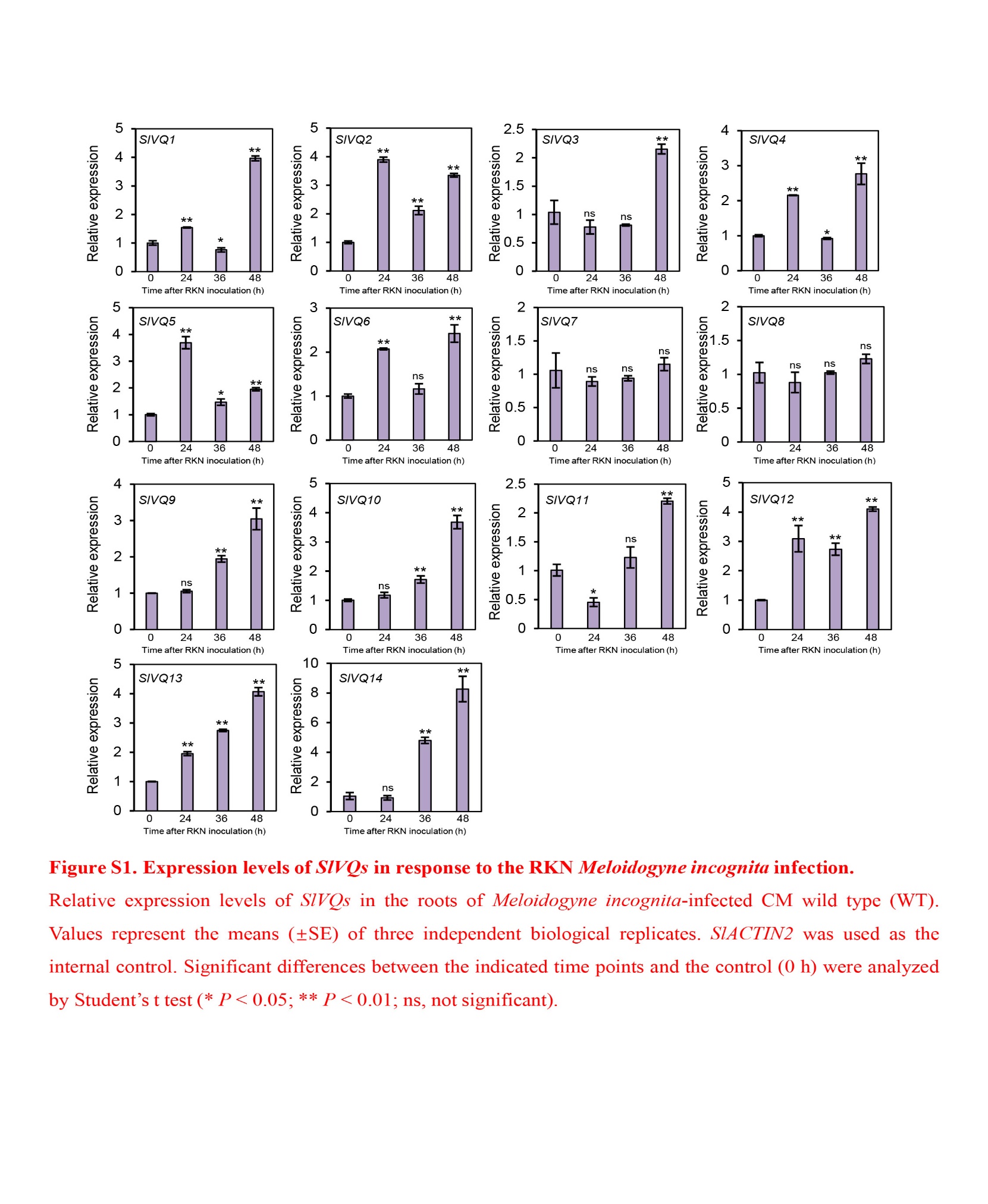


Figure S1. Expression levels of *SlVQs* in response to the RKN *Meloidogyne incognita* infection.
Relative expression levels of *SlVQs* in the roots of *Meloidogyne incognita*-infected CM wild type (WT). Values represent the means (±SE) of three independent biological replicates. *SlACTIN2* was used as the internal control. Significant differences between the indicated time points and the control (0 h) were analyzed by Student’s *t* test (* *P* < 0.05; ** *P* < 0.01; ns, not significant).


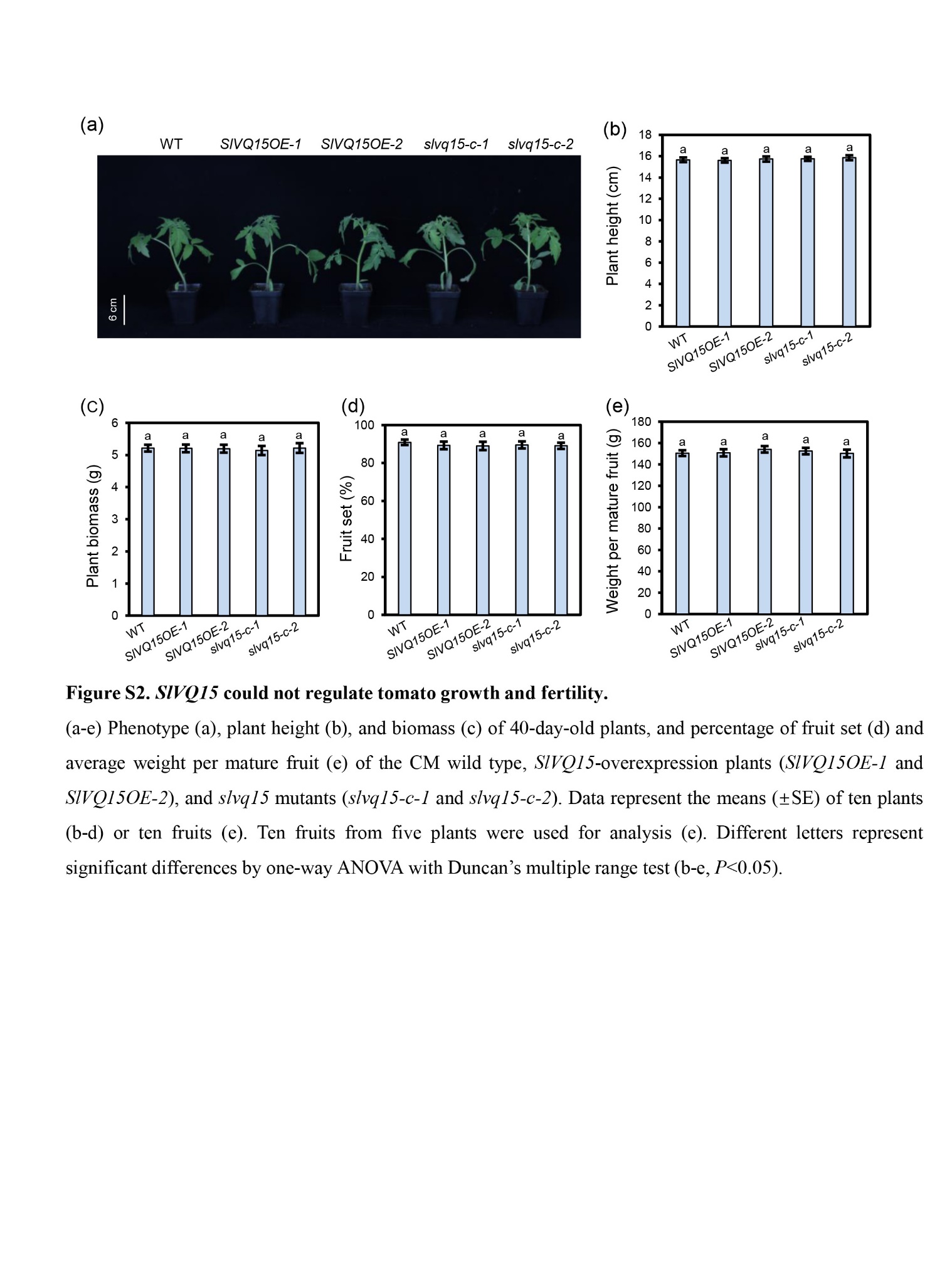


**Figure S2. *SlVQ15* could not regulate tomato growth and fertility.**

(a-e) Phenotype (a), plant height (b), and biomass (c) of 40-day-old plants, and percentage of fruit set (d) and average weight per mature fruit (e) of the CM wild type, *SlVQ15*-overexpression plants (*SlVQ15OE-1* and *SlVQ15OE-2*), and *slvq15* mutants (*slvq15-c-1* and *slvq15-c-2*). Data represent the means (±SE) of ten plants (b-d) or ten fruits (e). Ten fruits from five plants were used for analysis (e). Different letters represent significant differences by one-way ANOVA with Duncan’s multiple range test (b-e, *P*<0.05).


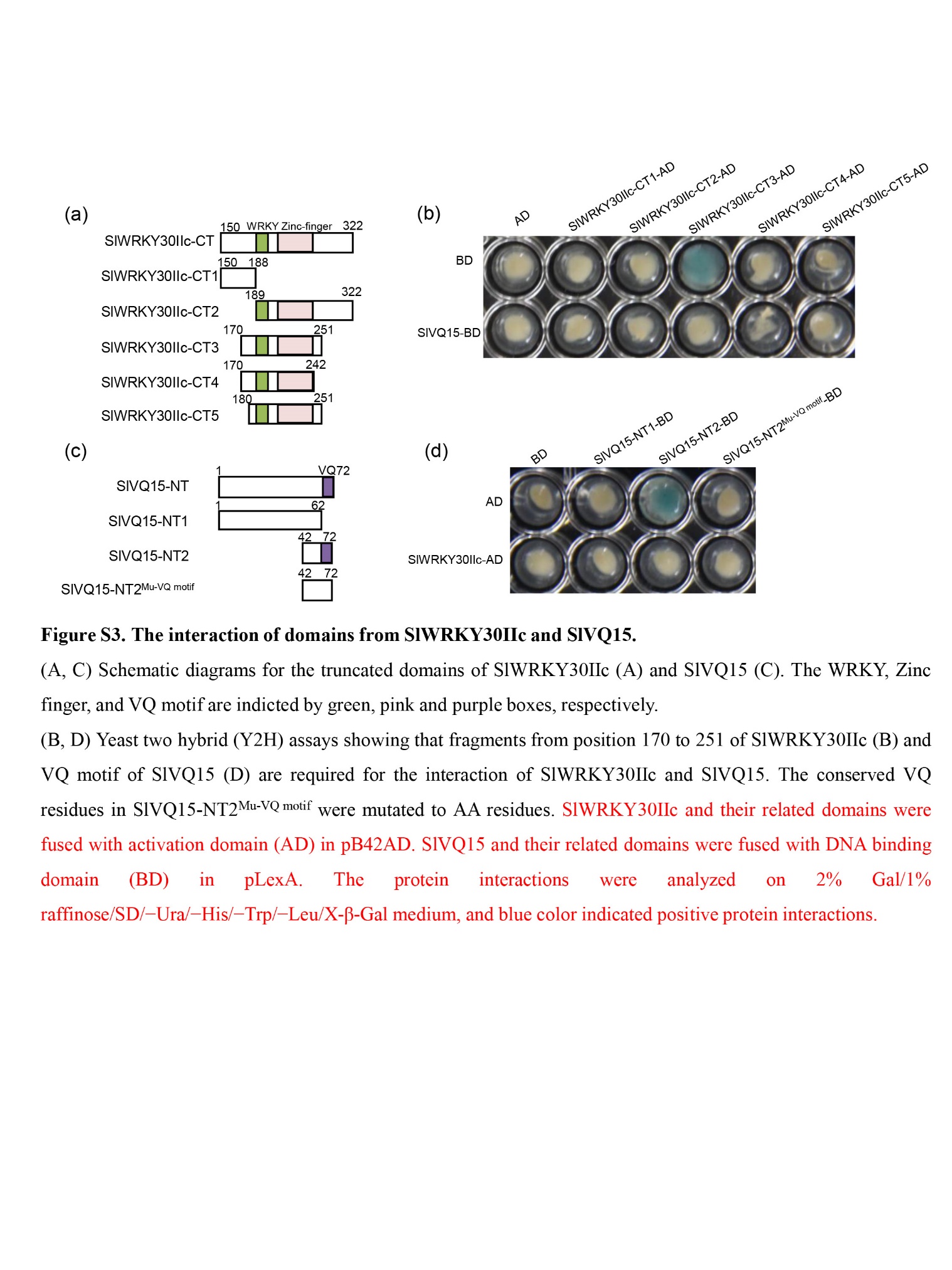


**Figure S3. The interaction of domains from SlWRKY30IIc and SlVQ15.**

(a, c) Schematic diagrams for the truncated domains of SlWRKY30IIc (a) and SlVQ15 (c). The WRKY, Zinc finger, and VQ motif are indicted by green, pink and purple boxes, respectively.

(b, d) Yeast two hybrid (Y2H) assays showing that fragments from position 170 to 251 of SlWRKY30IIc (b) and VQ motif of SlVQ15 (d) are required for the interaction of SlWRKY30IIc and SlVQ15. The conserved VQ residues in SlVQ15-NT2^Mu-VQ motif^ were mutated to AA residues. SlWRKY30IIc and their related domains were fused with activation domain (AD) in pB42AD. SlVQ15 and their related domains were fused with DNA binding domain (BD) in pLexA. The protein interactions were analyzed on 2% Gal/1% raffinose/SD/−Ura/−His/−Trp/−Leu/X-β-Gal medium, and blue color indicated positive protein interactions.


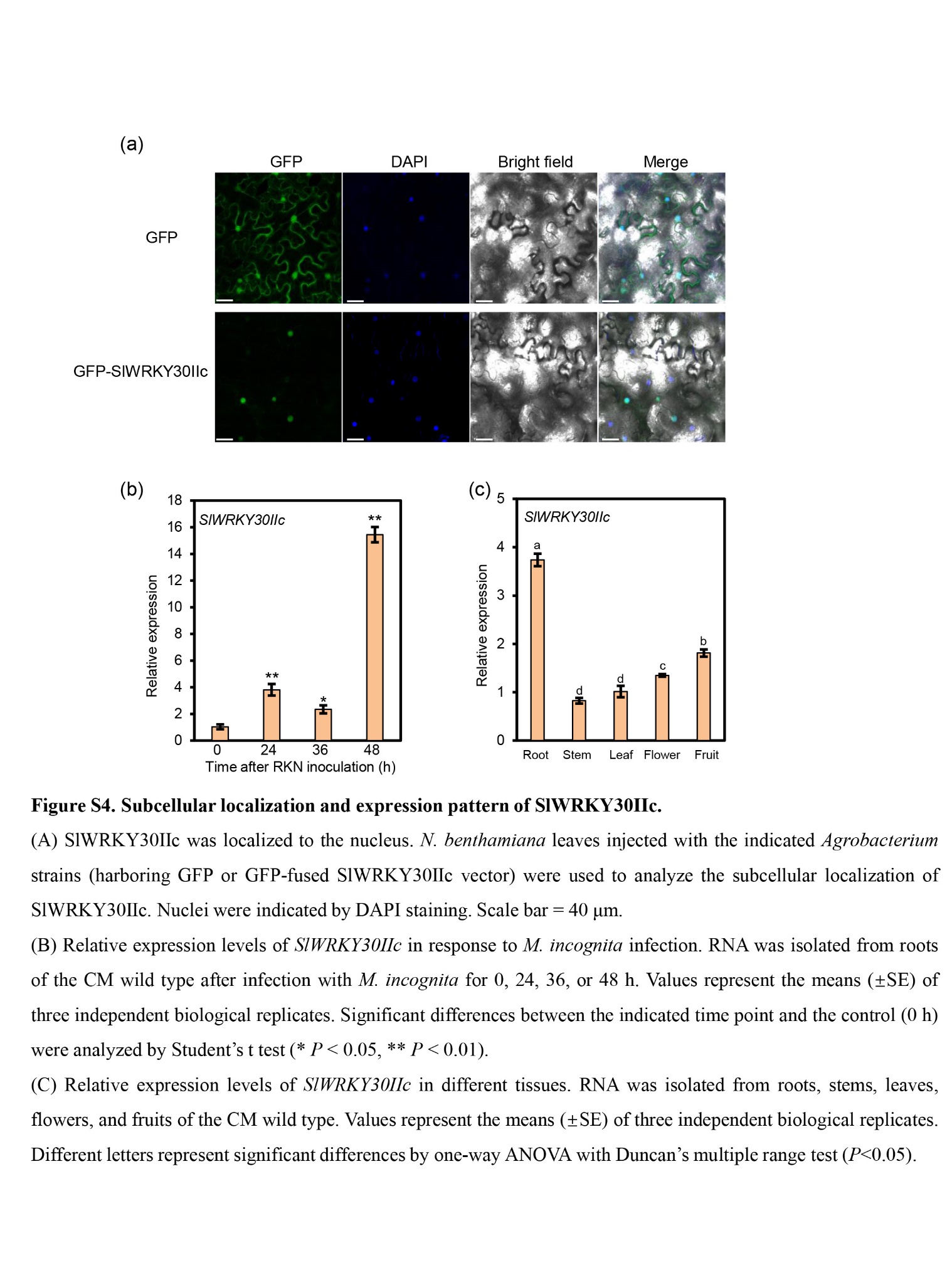


**Figure S4. Subcellular localization and expression pattern of SlWRKY30IIc.**

(a) SlWRKY30IIc was localized to the nucleus. *N. benthamiana* leaves injected with the indicated *Agrobacterium* strains (harboring GFP or GFP-fused SlWRKY30IIc vector) were used to analyze the subcellular localization of SlWRKY30IIc. Nuclei were indicated by DAPI staining. Scale bar = 40 μm.

(b) Relative expression levels of *SlWRKY30IIc* in response to *M. incognita* infection. RNA was isolated from roots of the CM wild type after infection with *M. incognita* for 0, 24, 36, or 48 h. Values represent the means (±SE) of three independent biological replicates. Significant differences between the indicated time point and the control (0 h) were analyzed by Student’s *t* test (* *P* < 0.05, ** *P* < 0.01).

(c) Relative expression levels of *SlWRKY30IIc* in different tissues. RNA was isolated from roots, stems, leaves, flowers, and fruits of the CM wild type. Values represent the means (±SE) of three independent biological replicates. Different letters represent significant differences by one-way ANOVA with Duncan’s multiple range test (*P*<0.05).


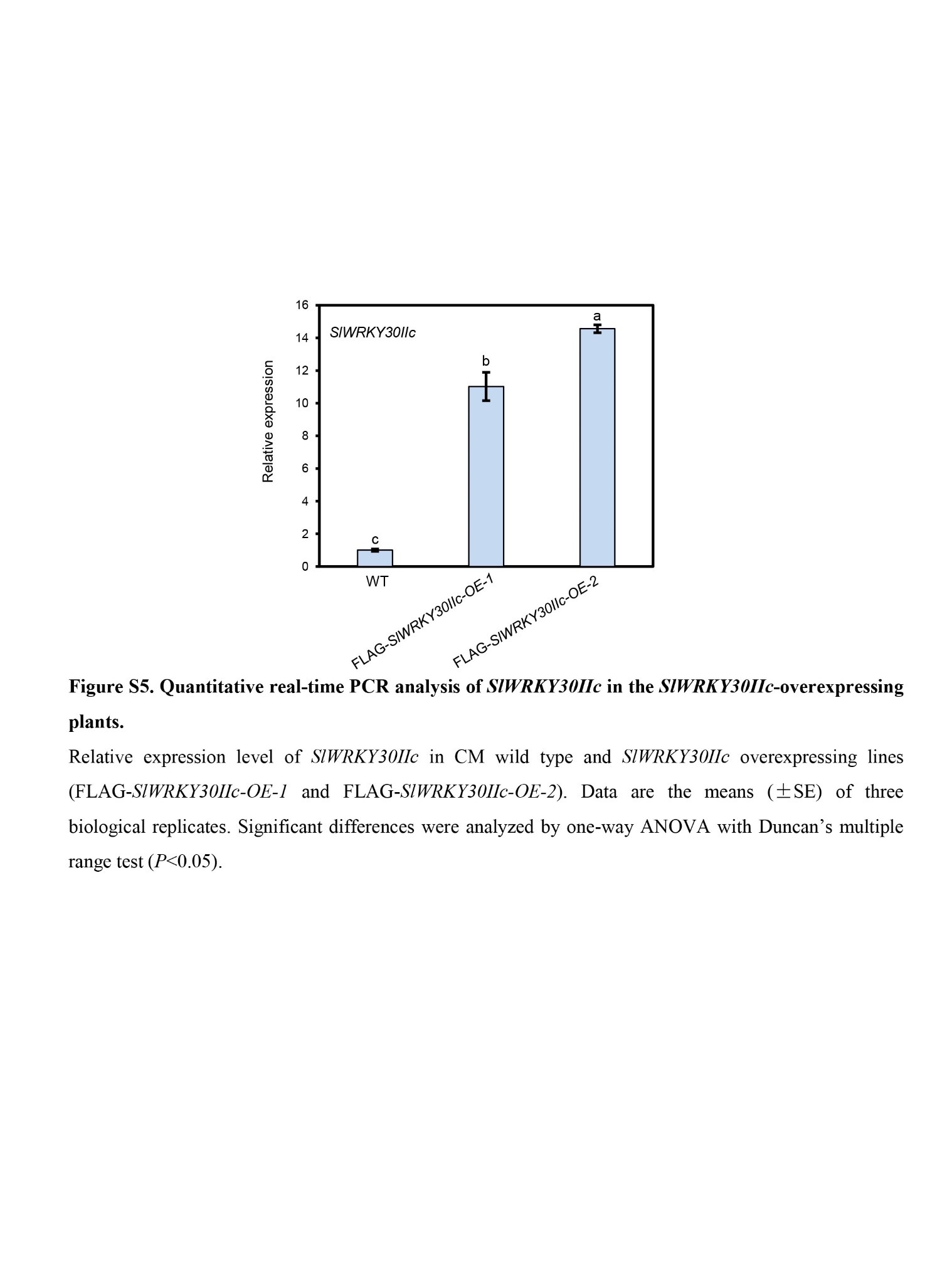


**Figure S5. Quantitative real-time PCR analysis of *SlWRKY30IIc* in the *SlWRKY30IIc*-overexpressing plants.**

Relative expression level of *SlWRKY30IIc* in CM wild type and *SlWRKY30IIc* overexpressing lines (FLAG-*SlWRKY30IIc-OE-1* and FLAG-*SlWRKY30IIc-OE-2*). Data are the means (±SE) of three biological replicates. Significant differences were analyzed by one-way ANOVA with Duncan’s multiple range test (*P*<0.05).


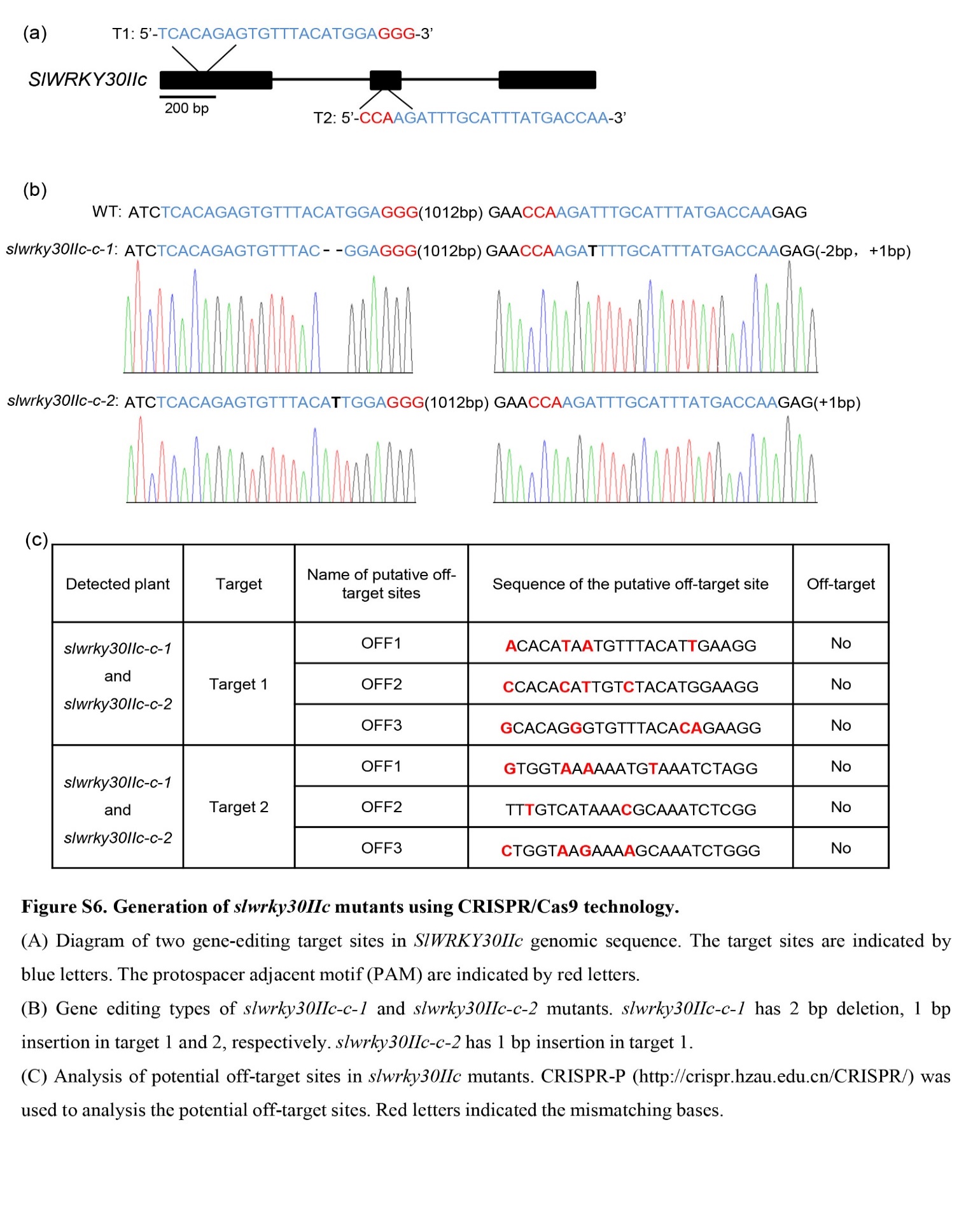


**Figure S6. Generation of *slwrky30IIc* mutants using CRISPR/Cas9 technology.**

(a) Diagram of two gene-editing target sites in *SlWRKY30IIc* genomic sequence. The target sites are indicated by blue letters. The protospacer adjacent motif (PAM) are indicated by red letters.

(b) Gene editing types of *slwrky30IIc-c-1* and *slwrky30IIc-c-2* mutants. *slwrky30IIc-c-1* has 2 bp deletion, 1 bp insertion in target 1 and 2, respectively. *slwrky30IIc-c-2* has 1 bp insertion in target 1.

(c) Analysis of potential off-target sites in *slwrky30IIc* mutants. CRISPR-P (http://crispr.hzau.edu.cn/CRISPR/) was used to analysis the potential off-target sites. Red letters indicated the mismatching bases.


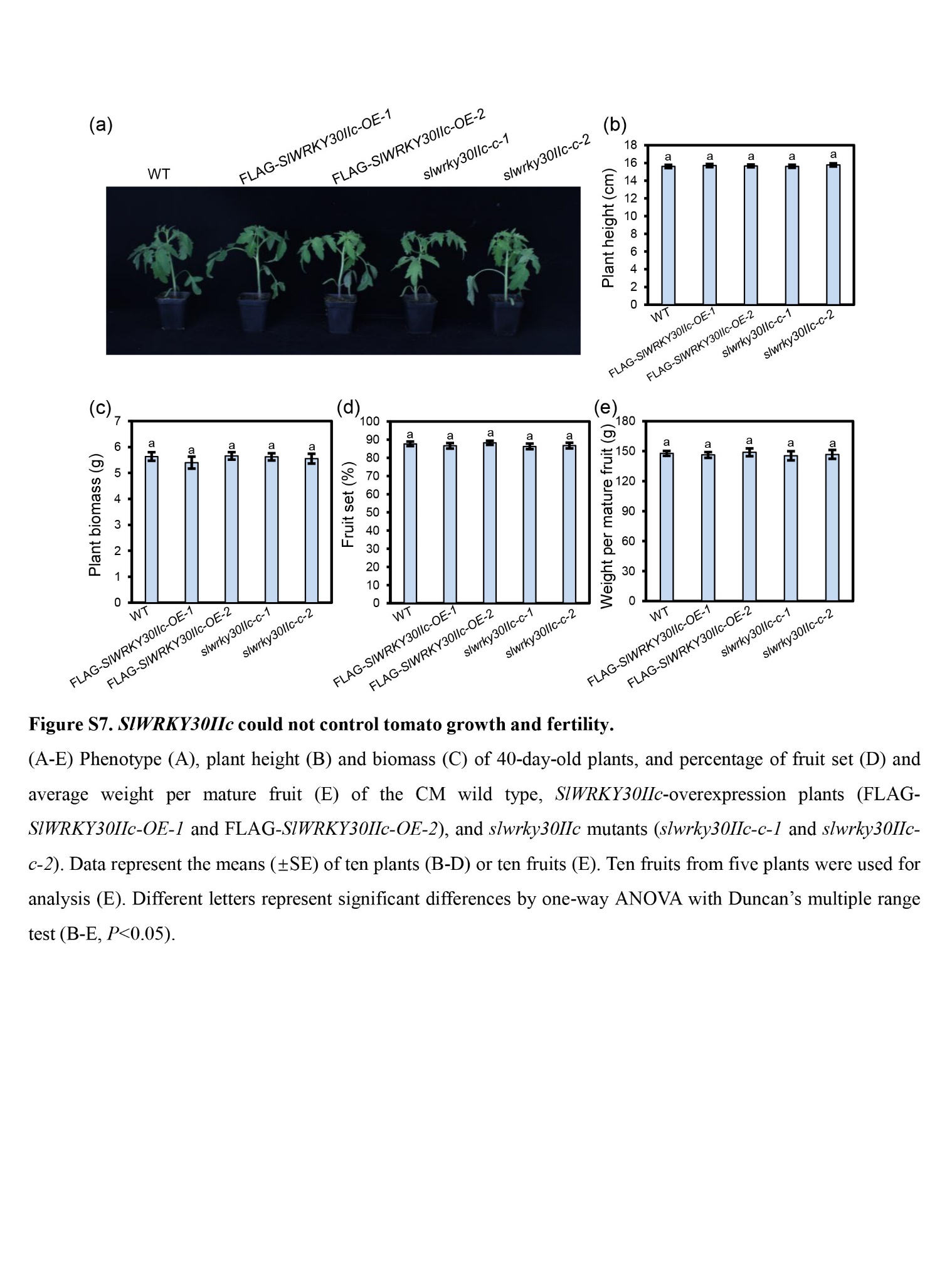


**Figure S7. *SlWRKY30IIc* could not control tomato growth and fertility.**

(a-e) Phenotype (a), plant height (b) and biomass (c) of 40-day-old plants, and percentage of fruit set (d) and average weight per mature fruit (e) of the CM wild type, *SlWRKY30IIc*-overexpression plants (FLAG-*SlWRKY30IIc-OE-1* and FLAG*-SlWRKY30IIc-OE-2*), and *slwrky30IIc* mutants (*slwrky30IIc-c-1* and *slwrky30IIc-c-2*). Data represent the means (±SE) of ten plants (b-d) or ten fruits (e). Ten fruits from five plants were used for analysis (e). Different letters represent significant differences by one-way ANOVA with Duncan’s multiple range test (b-e, *P*<0.05).


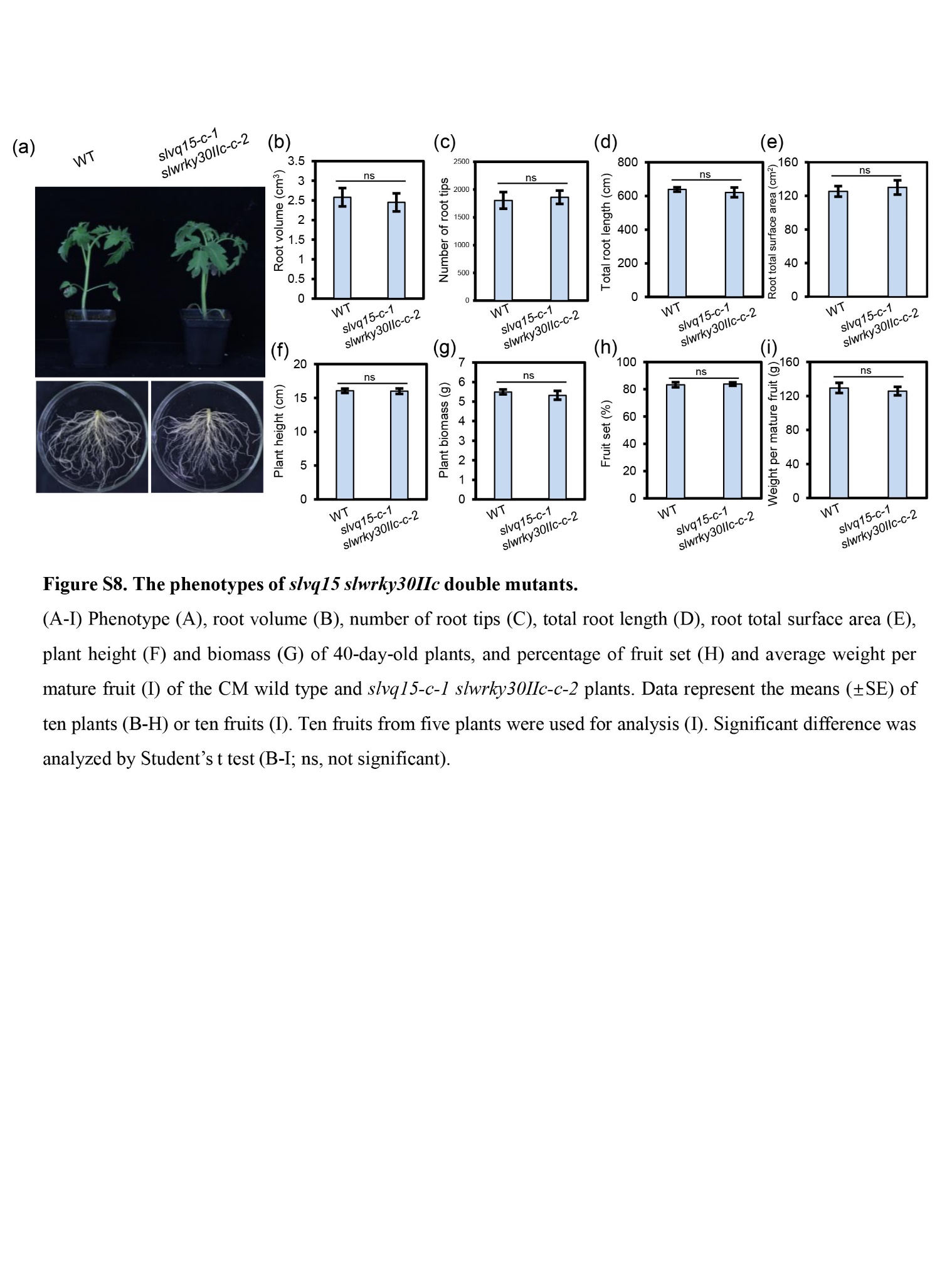


**Figure S8. The phenotypes of *slvq15* *slwrky30IIc* double mutants.**

(a-i) Phenotype (a), root volume (b), number of root tips (c), total root length (d), root total surface area (e), plant height (f) and biomass (g) of 40-day-old plants, and percentage of fruit set (h) and average weight per mature fruit (i) of the CM wild type and *slvq15-c-1 slwrky30IIc-c-2* plants. Data represent the means (±SE) of ten plants (b-h) or ten fruits (i). Ten fruits from five plants were used for analysis (i). Significant difference was analyzed by Student’s *t* test (b-i; ns, not significant).


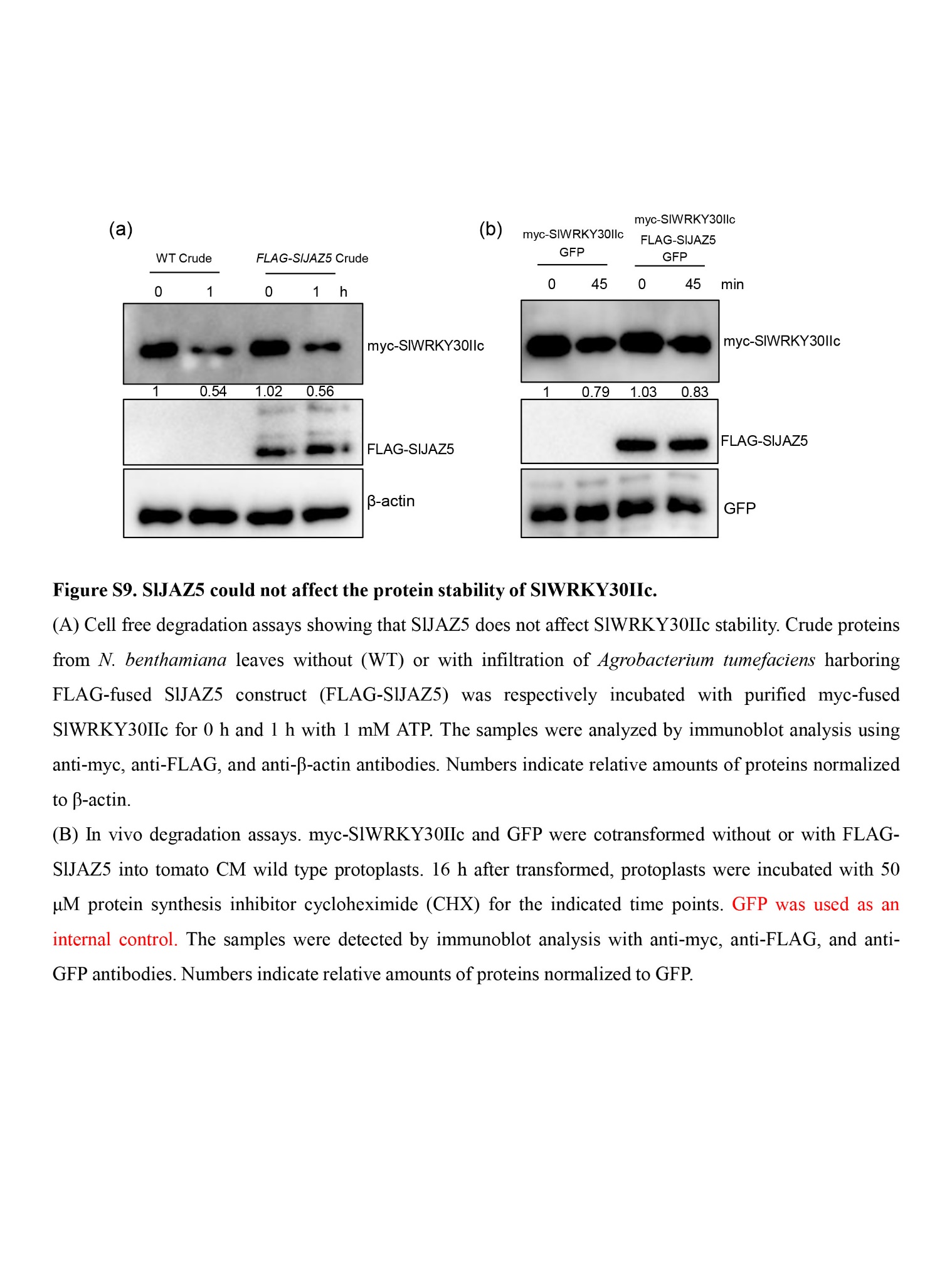


**Figure S9. SlJAZ5 could not affect the protein stability of SlWRKY30IIc.**

(a) Cell free degradation assays showing that SlJAZ5 does not affect SlWRKY30IIc stability. Crude proteins from *N. benthamiana* leaves without (WT) or with infiltration of *Agrobacterium tumefaciens* harboring FLAG-fused SlJAZ5 construct (FLAG-SlJAZ5) was respectively incubated with purified myc-fused SlWRKY30IIc for 0 h and 1 h with 1 mM ATP. The samples were analyzed by immunoblot analysis using anti-myc, anti-FLAG, and anti-β-actin antibodies. Numbers indicate relative amounts of proteins normalized to β-actin.

(b) In vivo degradation assays. myc-SlWRKY30IIc and GFP were cotransformed without or with FLAG-SlJAZ5 into tomato CM wild type protoplasts. 16 h after transformed, protoplasts were incubated with 50 μM protein synthesis inhibitor cycloheximide (CHX) for the indicated time points. GFP was used as an internal control. The samples were detected by immunoblot analysis with anti-myc, anti-FLAG, and anti-GFP antibodies. Numbers indicate relative amounts of proteins normalized to GFP.


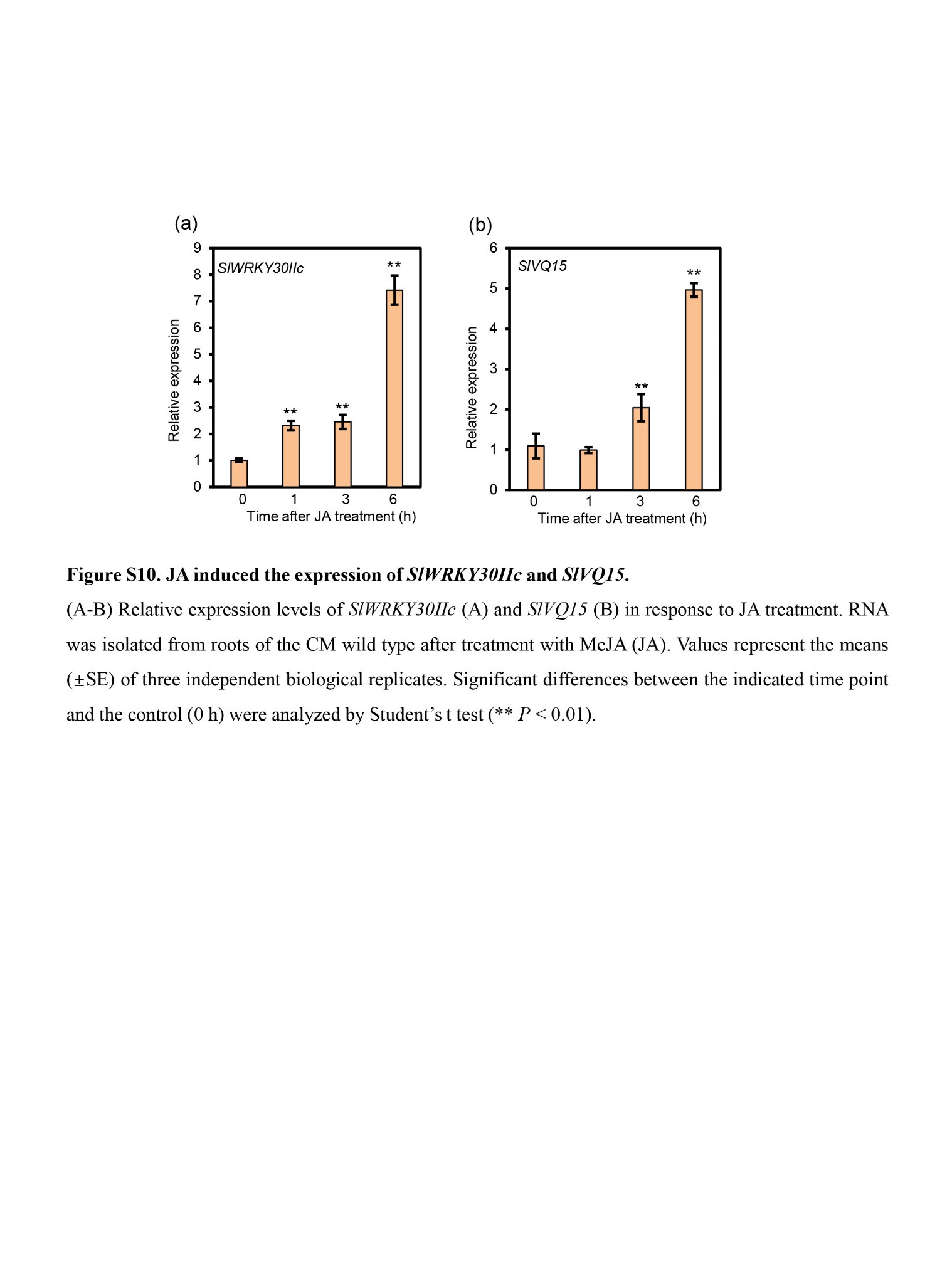


**Figure S10. JA induced the expression of *SlWRKY30IIc* and *SlVQ15*.**

(a-b) Relative expression levels of *SlWRKY30IIc* (a) and *SlVQ15* (b) in response to JA treatment. RNA was isolated from roots of the CM wild type after treatment with MeJA (JA). Values represent the means (±SE) of three independent biological replicates. Significant differences between the indicated time point and the control (0 h) were analyzed by Student’s t test (** *P* < 0.01).


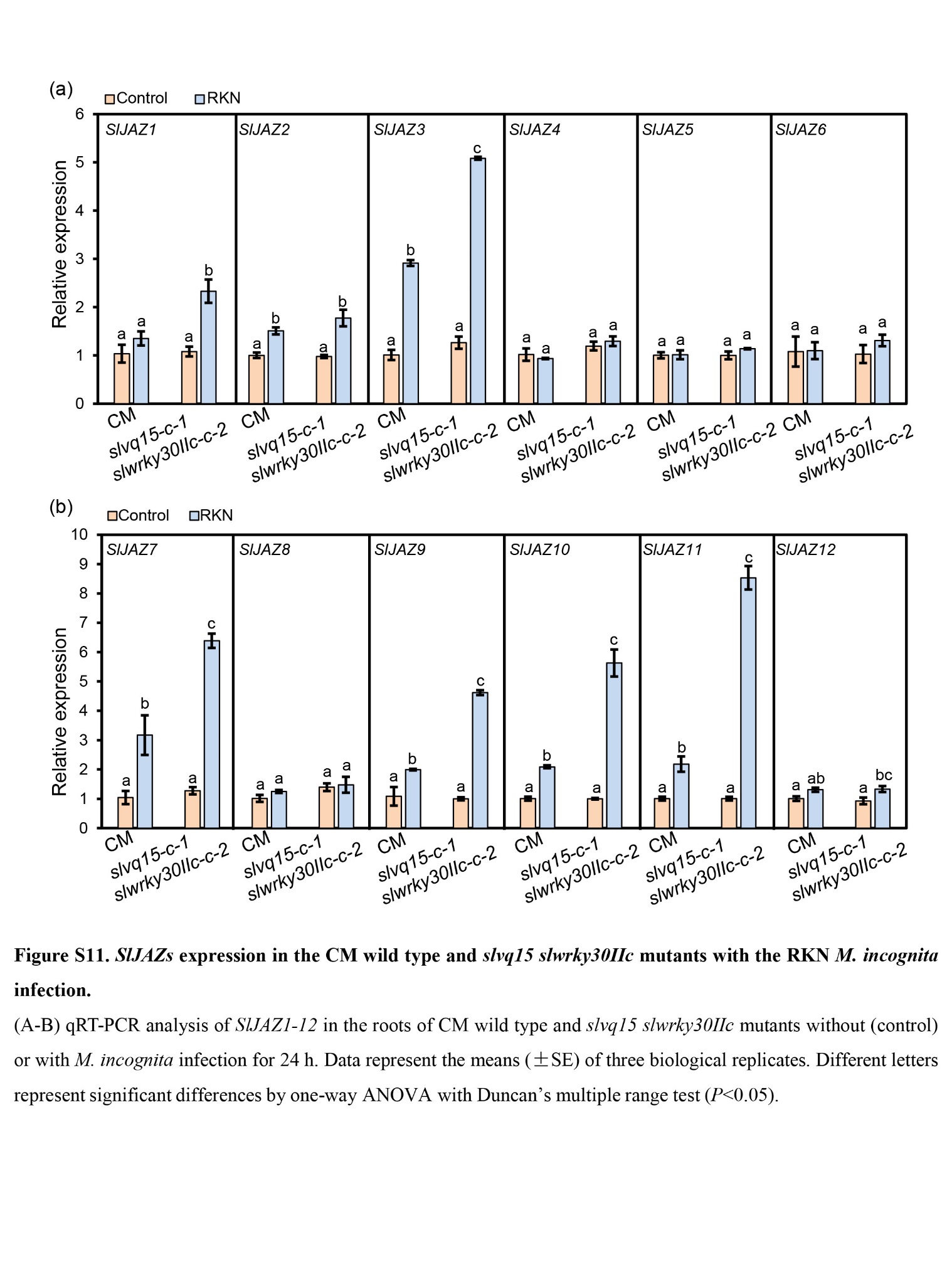


**Figure S11. *SlJAZs* expression in the CM wild type and *slvq15 slwrky30IIc* mutants with the RKN *M. incognita* infection.**

(a-b) qRT-PCR analysis of *SlJAZ1-12* in the roots of CM wild type and *slvq15 slwrky30IIc* mutants without (control) or with *M. incognita* infection for 24 h. Data represent the means (±SE) of three biological replicates. Different letters represent significant differences by one-way ANOVA with Duncan’s multiple range test (*P*<0.05).


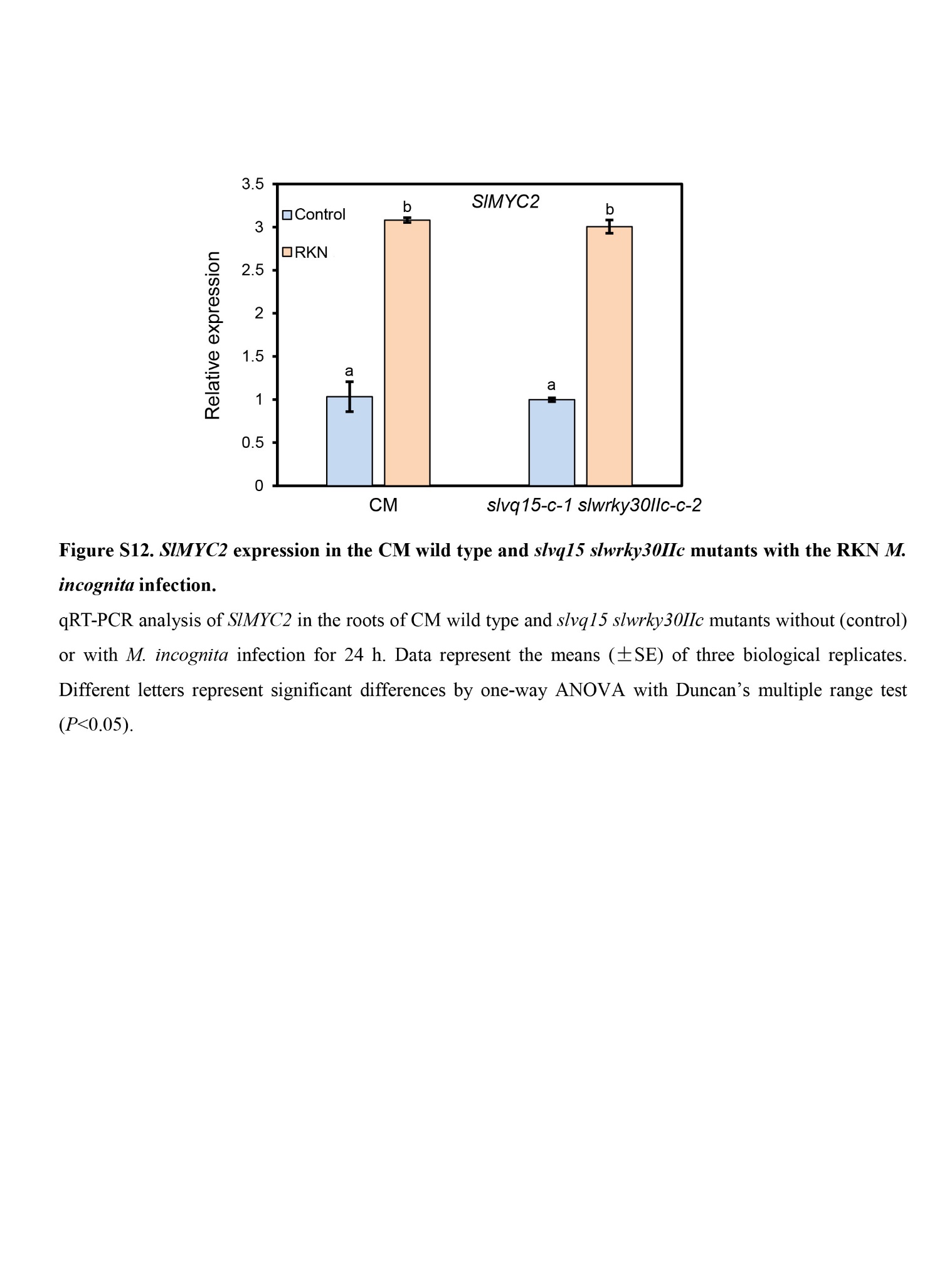


**Figure S12. *SlMYC2* expression in the CM wild type and *slvq15 slwrky30IIc* mutants with the RKN *M. incognita* infection.**

qRT-PCR analysis of *SlMYC2* in the roots of CM wild type and *slvq15 slwrky30IIc* mutants without (control) or with *M. incognita* infection for 24 h. Data represent the means (±SE) of three biological replicates. Different letters represent significant differences by one-way ANOVA with Duncan’s multiple range test (*P*<0.05).


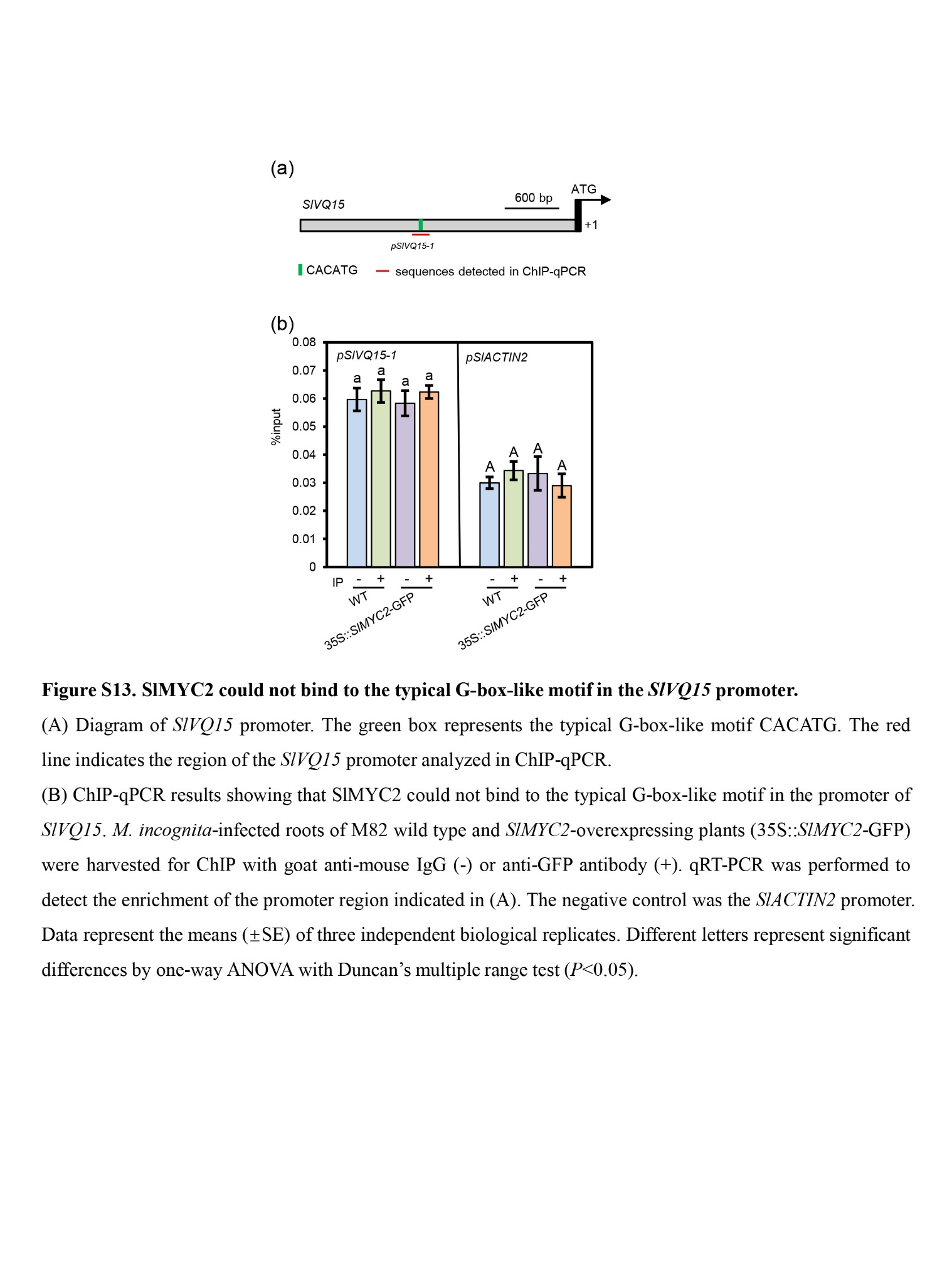


**Figure S13. SlMYC2 could not bind to the typical G-box-like motif in the *SlVQ15* promoter*.***

(a) Diagram of *SlVQ15* promoter. The green box represents the typical G-box-like motif CACATG. The red line indicates the region of the *SlVQ15* promoter analyzed in ChIP-qPCR.

(b) ChIP-qPCR results showing that SlMYC2 could not bind to the typical G-box-like motif in the promoter of *SlVQ15*. *M. incognita*-infected roots of M82 wild type and *SlMYC2*-overexpressing plants (35S::*SlMYC2*-GFP) were harvested for ChIP with goat anti-mouse IgG (-) or anti-GFP antibody (+). qRT-PCR was performed to detect the enrichment of the promoter region indicated in (A). The negative control was the *SlACTIN2* promoter. Data represent the means (±SE) of three independent biological replicates. Different letters represent significant differences by one-way ANOVA with Duncan’s multiple range test (*P*<0.05).


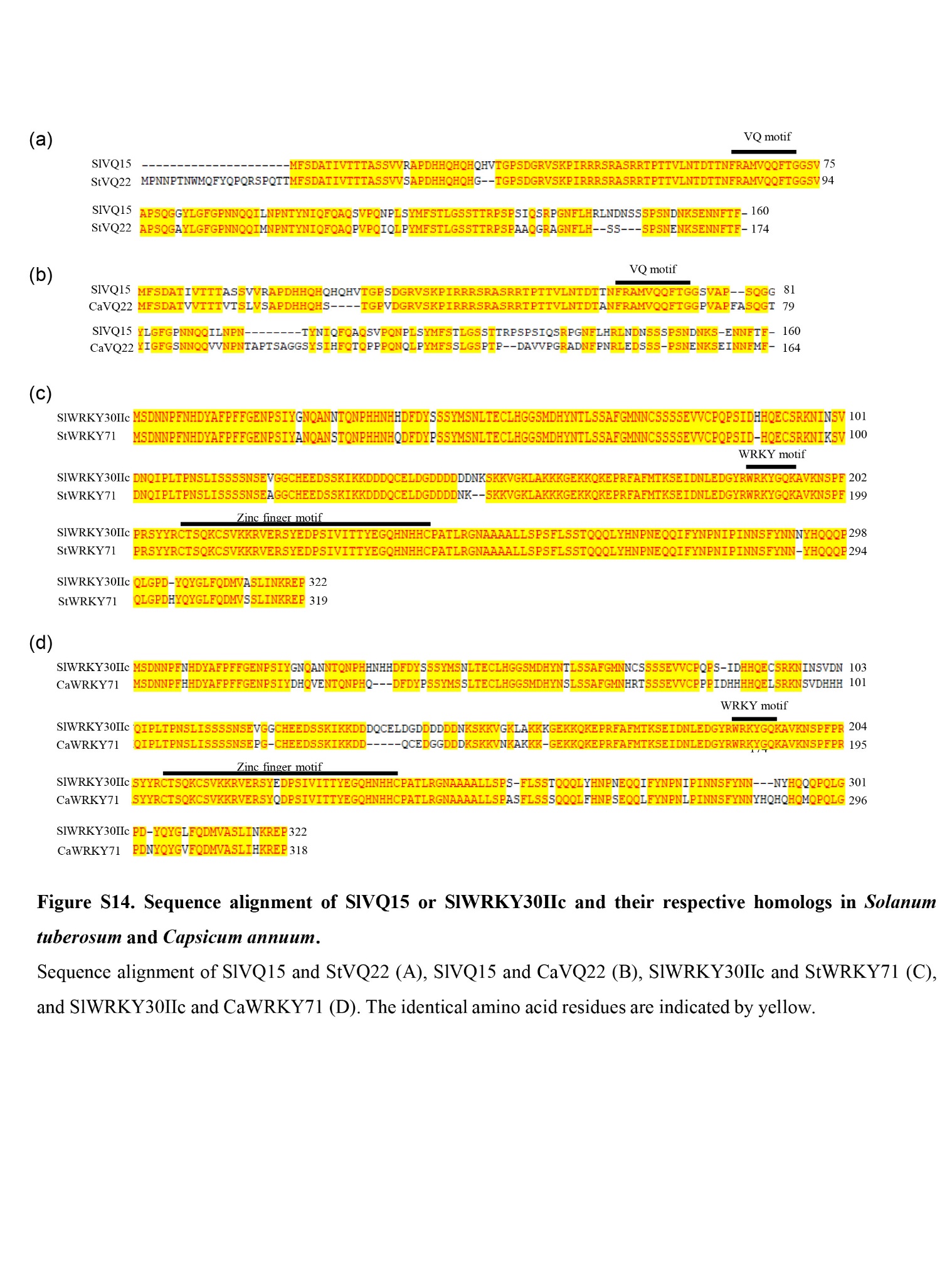


**Figure S14. Sequence alignment of SlVQ15 or SlWRKY30IIc and their respective homologs in *Solanum tuberosum* and *Capsicum annuum*.**

Sequence alignment of SlVQ15 and StVQ22 (a), SlVQ15 and CaVQ22 (b), SlWRKY30IIc and StWRKY71 (c), and SlWRKY30IIc and CaWRKY71 (d). The identical amino acid residues are indicated by yellow.
