# Supplementary material for: SlVQ15 recruits SlWRKY30IIc to link with jasmonate pathway in regulating tomato defence against root‐knot nematodes
Source: Plant Biotechnol J. 2024 Nov 5;23(1):235–49. doi: 10.1111/pbi.14493 (PMC11672745; doi:10.1111/pbi.14493)
Supplement: Supplementary file 2 — Table S1 Positive clones of SlVQ15 interaction candidates in Y2H screening assay. Table S2 Primers used for vector construction. Table S3 Primers used for analysis of the target site mutation. Table S4 Primers used for analysis of the existence of Cas9. Table S5 Primers used for analysis of off‐target site mutation. Table S6 Primers used for qRT‐PCR analysis. [file PBI-23-235-s002.docx]

**Table S1.** **Positive clones of SlVQ15-interaction candidates in Y2H screening assay.**

| **Sol No.** | **Frequency** | **Description** |
| --- | --- | --- |
| Solyc01g066840.2.1 | 2 | 40S ribosomal protein S21-2 |
| Solyc01g096510.2.1 | 1 | Sigma factor binding protein 1 |
| Solyc01g104920.2.1 | 1 | 26S protease regulatory subunit 8 homologs |
| Solyc01g110020.2.1 | 2 | BAH-PHD domain-containing protein |
| Solyc02g085950.2.1 | 1 | ribulose bisphosphate carboxylase small chain 3B |
| Solyc02g089470.1.1 | 1 | unknown Protein |
| Solyc03g034220.2.1 | 7 | Tomato RuBP carboxylase small subunit |
| Solyc03g006640.2.1 | 1 | F-box family protein |
| Solyc03g115110.2.1 | 1 | ATP synthase gamma chain |
| Solyc03g116140.2.1 | 3 | Activating signal cointegrator 1 |
| Solyc04g057940.2.1 | 2 | U-box domain-containing protein |
| Solyc04g080700.2.1 | 1 | Wound responsive protein |
| Solyc04g011580.2.1 | 2 | mRNA binding protein Pumilio 2 |
| Solyc05g007510.2.1 | 1 | RNA-dependent RNA polymerase |
| Solyc05g008010.2.1 | 3 | 60S ribosomal protein L31 |
| Solyc05g012500.2.1 | 1 | WRKY transcription factor 57 |
| Solyc05g053380.2.1 | 1 | WRKY transcription factor 48 |
| Solyc06g005060.2.1 | 2 | Elongation factor 1-alpha |
| Solyc06g005170.2.1 | 2 | Mitogen-activated protein kinase 3 |
| Solyc06g076020.2.1 | 1 | Heat shock protein 70 kD |
| Solyc06g083980.1.1 | 1 | bHLH transcription factor138 |
| Solyc07g006790.2.1 | 1 | Dihydrolipoyllysine-residue acetyltransferase component of pyruvate dehydrogenase complex |
| Solyc07g056280.2.1 | 4 | WRKY transcription factor 30 |
| Solyc07g064100.1.1 | 1 | Unknown Protein |
| Solyc08g006900.2.1 | 1 | Ribosomal protein L32 |
| Solyc08g082820.2.1 | 3 | TOMBIPGRBC Tomato BiP (binding protein) |
| Solyc09g008800.2.1 | 4 | 60S ribosomal protein L24 |
| Solyc09g061860.2.1 | 2 | Sterol 3-beta-glucosyltransferase |
| Solyc09g082520.2.1 | 3 | 40S ribosomal protein S1 |
| Solyc10g044680.1.1 | 1 | R2R3MYB transcription factor 55 |
| Solyc10g078440.1.1 | 2 | VQ-motif containing protein 22 |
| Solyc10g079070.1.1 | 1 | bHLH transcription factor 65 |
| Solyc11g009060.1.1 | 1 | Unknown Protein |
| Solyc11g010850.1.1 | 1 | 1-deoxy-D-xylulose 5-phosphate synthase 2 |
| Solyc12g010040.1.1 | 3 | 60S ribosomal protein L10 |
| Solyc12g094640.1.1 | 2 | Glyceraldehyde-3-phosphate dehydrogenase B |
| Solyc12g099990.1.1 | 1 | Calmodulin 2 |

**Table S2. Primers used for vector construction.**

| **Assay** | **Primer** | **Sequence (5’-3’)** |
| --- | --- | --- |
| **Y2H** | SlWRKY30IIc-AD-F | GGGGAATTCATGTCTGATAATAACCCTTTTAATC |
|  | SlWRKY30IIc-AD-R | CCGCTCGAGTCATGGCTCTCTTTTGTTGATC |
|  | SlWRKY30IIc-NT-AD-F | GGGGAATTCATGTCTGATAATAACCCTTTTAATC |
|  | SlWRKY30IIc-NT-AD-R | CCGCTCGAGTTACTTTGCCAATTTCCCCACTTTC |
|  | SlWRKY30IIc-CT-AD-F | CCCCCCATGGATGTGATGATAATAAGTCTAAGAAAG |
|  | SlWRKY30IIc-CT-AD-R | CCGCTCGAGTCATGGCTCTCTTTTGTTGATC |
|  | SlWRKY30IIc-CT1-AD-F | CCCCCCATGGATGTGATGATAATAAGTCTAAGAA |
|  | SlWRKY30IIc-CT1-AD-R | CCGCTCGAGTCACCATCTTCAAGATTATCAATCT |
|  | SlWRKY30IIc-CT2-AD-F | CCCCCCATGGATGCTATAGATGGAGAAAATATGG |
|  | SlWRKY30IIc-CT2-AD-R | CCGCTCGAGTCATGGCTCTCTTTTGTTGATC |
|  | SlWRKY30IIc-CT3-AD-F | CCCCCCATGGATGCATTTATGACCAAGAGTGAGA |
|  | SlWRKY30IIc-CT3-AD-R | CCGCTCGAGTCAAGCAGCATTTCCTCTAAGAGTA |
|  | SlWRKY30IIc-CT4-AD-F | CCCCCCATGGATGCATTTATGACCAAGAGTGAGA |
|  | SlWRKY30IIc-CT4-AD-R | CCGCTCGAGTCAACAATGATGGTTATGTTGGCC |
|  | SlWRKY30IIc-CT5-AD-F | CCCCCCATGGATGAGTGAGATTGATAATCTTGAAG |
|  | SlWRKY30IIc-CT5-AD-R | CCGCTCGAGTCAAGCAGCATTTCCTCTAAGAGTA |
|  | SlVQ15-BD-F | GGGGAATTCATGTTCTCCGATGCCACAATTG |
|  | SlVQ15-BD -R | CCGCTCGAGTTAGAAGGTGAAATTATTTTCCG |
|  | SlVQ15-NT-BD-F | GGGGAATTCATGTTCTCCGATGCCACAATTG |
|  | SlVQ15-NT-BD -R | CCGCTCGAGTTAACCAGTAAACTGTTGCACCATC |
|  | SlVQ15-NT1-BD-F | GGGGAATTCATGTTCTCCGATGCCACAATTG |
|  | SlVQ15-NT1-BD -R | CCGCTCGAGTTAATTAGTTGTATCCGTGTTTA |
|  | SlVQ15-NT2-BD-F | GGGGAATTCATGTTCCGTGCGATGGTGCAACAGTT |
|  | SlVQ15-NT2-BD -R | CCGCTCGAGTTAAATTTGCTGATTGTTGGGCCC |
|  | SlVQ15-NT2 △VQ-motif -BD-F | CACCATCACAAGGGGGCTACTACTACTTTG |
|  | SlVQ15-NT2 △VQ-motif -BD -R | GATTGTTGGGCCCAAAGTAGTAGTAG |
| **LCI** | SlVQ15-nLUC-F | GAGAACACGGGGGACGAGCTCGGTACCATGTTCTCCGATGCCACAATTG |
|  | SlVQ15-nLUC-R | GGTCGAGTGAGGAGAAGAGCCGGGCCCCGAAGGTGAAATTATTTTCCG |
|  | SlVQ15-NT-nLUC-F | CGGGGTACCATGTTCTCCGATGCCACAATTG |
|  | SlVQ15-NT-nLUC-R | ACGCGTCGACACCAGTAAACTGTTGCACCATC |
|  | cLUC-SlWRKY30IIc-F | CGGGGTACCATGTCTGATAATAACCCTTTTAATC |
|  | cLUC-SlWRKY30IIc-R | ACGCGTCGACTCATGGCTCTCTTTTGTTGATC |
|  | cLUC-SlWRKY30IIc-CT-F | CGGGGTACCATGTGATGATAATAAGTCTAAGAAAG |
|  | cLUC-SlWRKY30IIc-CT-R | ACGCGTCGACTCATGGCTCTCTTTTGTTGATC |
|  | SlJAZ1-nLUC-F | CGGGGTACCATGGCTTCATCGGAGATTGTGG |
|  | SlJAZ1-nLUC-R | ACGCGTCGACGTATTGCTCAGTTTTCACT |
|  | SlJAZ3-nLUC-F | CGGGGTACCATGTCGAATTTATGTGACGCTC |
|  | SlJAZ3-nLUC-R | ACGCGTCGACTAACTTGAAATTGAGATC |
|  | SlJAZ5-nLUC-F | CGGGGTACCATGGAGAGAGATTTCATGGGGTTG |
|  | SlJAZ5-nLUC-R | ACGCGTCGACCTTGACCAAACTGATTATG |
|  | 35S-SlJAZ5-F | ACGCGTCGACATGGAGAGAGATTTCATGGGGTTG |
|  | 35S-SlJAZ5-R | CGGACTAGTCTACTTGACCAAACTGATTATG |
| **Pull down** | flag-SlWRKY30IIc-F | ACGCGTCGACATGTCTGATAATAACCCTTTTAATC |
|  | flag-SlWRKY30IIc-R | CGGACTAGTTCATGGCTCTCTTTTGTTGATC |
|  | MBP-SlVQ15-F | ACGCGTCGACATGTTCTCCGATGCCACAATTG |
|  | MBP-SlVQ15-R | CCGGAATTCTTAGTGGTGGTGGTGGTGGTGGAAGGTGAAATTATTTTCCG |
| **Co-IP** | flag-SlJAZ5-F | ACGCGTCGACATGGAGAGAGATTTCATGGGGTTG |
|  | flag-SlJAZ5-R | CGGACTAGTCTACTTGACCAAACTGATTATG |
|  | GFP-SlWRKY30IIc -F | GGGGAATTCATGTCTGATAATAACCCTTTTAATC |
|  | GFP-SlWRKY30IIc- R | AGACCCGGGATGTCTGATAATAACCCTTTTAATC |
| **Subcellular**  **localization** | GFP-SlWRKY30IIc -F | GGGGAATTCATGTCTGATAATAACCCTTTTAATC |
|  | GFP-SlWRKY30IIc- R | AGACCCGGGATGTCTGATAATAACCCTTTTAATC |
| **Protein degradation** | myc-SlWRKY30IIc-F | GACTTGAATTCAAGGCCTCTCGAGCCCGGG ATGTCTGATAATAACCCTTTT |
|  | myc-SlWRKY30IIc-R | AATGTTTGAACGATCGGGGAAATTCGAGCTC TCATGGCTCTCTTTTGTT |
| **Dual-LUC** | SlWRKY30IIc-pGreenII 62-SK-F | CGCGGATCCATGTCTGATAATAACCCTTTTAATC |
|  | SlWRKY30IIc-pGreenII 62-SK-R | GGGGAATTCTCATGGCTCTCTTTTGTTGATC |
|  | SlMYC2-pGreenII 62-SK-F | CGCGGATCCATGACTGAATACAGCTTGCCCA |
|  | SlMYC2-pGreenII 62-SK-R | ATCCTGCAGTTAGTGTGTTTCAGCAATTTTCG |
|  | GFP-pGreenII 62-SK-F | CGCGGATCCATGGTGAGCAAGGGCGAGGAGC |
|  | GFP-pGreenII 62-SK-R | GGGGAATTCTTACTTGTACAGCTCGTCCATGC |
|  | SlJAZ5-pGreenII 62-SK-F | CGCGGATCCATGGTGAGCAAGGGCGAGGAGC |
|  | SlJAZ5-pGreenII 62-SK-R | ATCCTGCAGTTACTTGTACAGCTCGTCCATGC |
|  | SlWRKY30IIc promoter-pGREEN0800-F | CGCGGATCCTTGATCAATAAATATGATTTA |
|  | SlWRKY30IIc promoter-pGREEN0800-R | CCCCCCATGGATGGGATATTGAGGTACACAAC |
|  | SlJAZ3pro-pGREEN0800-F | ATCCTGCAGGGATTTAATAGGAATCTTGGTTA |
|  | SlJAZ3pro-pGREEN0800-R | CGCGGATCCTGTAGTAGTCGGCAACTGCGGCTAC |
|  | SlJAZ7pro-pGREEN0800-F | ATCCTGCAGCGTATGCTGGTTATATTAGTATAA |
|  | SlJAZ7pro-pGREEN0800-R | CGCGGATCCGAGAAGCTTCATGCAATAAGAAATAC |
|  | SlJAZ9pro-pGREEN0800-F | ATCCTGCAGTGTACACATGAGTGTTAGTGATCGG |
|  | SlJAZ9pro-pGREEN0800-R | CCCCCCATGGAAAGGGAAACTAGAATGAAT |
|  | SlJAZ11pro-pGREEN0800-F | ATCCTGCAGGAACAACAGATGATAAGTTTATAT |
|  | SlJAZ11pro-pGREEN0800-R | CGCGGATCCGCCAGCCATCACTTTCCGAAAGT |
|  | SlVQ15-pGreenII 62-SK-F | CGCGGATCCATGTTCTCCGATGCCACAATTG |
|  | SlVQ15-pGreenII 62-SK-R | GGGGAATTCTTAGAAGGTGAAATTATTTTCCG |
| **Construction of CRISPR/Cas9 vector** | SlWRKY30IIc-T1-F | CAATGGTCTCATGATTTCACAGAGTGTTTACATGGAGTTTTAGAGCTAGAAATA |
|  | SlWRKY30IIc-T2-R | CAATGGTCTCAATTGTGGTCATAAATGCAAATCTGTTTTAGAGCTAGAAATAG |
| **Construction of SlWRKY30IIc-overexpression vector** | flag-SlWRKY30IIc-OE-F | ACGCGTCGACATGTCTGATAATAACCCTTTTAATC |
|  | flag-SlWRKY30IIc-OE-R | CGGACTAGTTCATGGCTCTCTTTTGTTGATC |

**Table S3. Primers used for analysis of the target site mutation.**

| **Target site** | **Primer** | **Sequence (5’-3’)** |
| --- | --- | --- |
| SlWRKY30IIc-T1 | For | CACAAAACCCTCATCATAATCATC |
|  | Rev | TCCACCAACCTCACTATTAGAAGA |
| SlWRKY30IIc-T2 | For | TTGGCAAAGAAGAAAGGAGAAAAG |
|  | Rev | GCTTGTTAATCCATTTCTAATAGTA |

**Table S4. Primers used for analysis of the existence of *Cas9*.**

| **Primer** | **Sequence (5’-3’)** |
| --- | --- |
| Cas9-F | GACAAGAAGTACAGCATCGG |
| Cas9-R | CTCGTGGTAGGCCACCTCG |

**Table S5. Primers used for analysis of off-target site mutation.**

| **Off-target site** | **Primer** | **Sequence (5’-3’)** |
| --- | --- | --- |
| SlWRKY30IIc-target 1  off-target site 1 | For | TCTTGTCTTAACATGTTAAAGC |
|  | Rev | AGGTTGTTCATGTTGCCAATGGT |
| SlWRKY30IIc-target 1  off-target site 2 | For | TCATGGCTCACCATCAGGTTC |
|  | Rev | GAAGAGATTAACTCAATTGAG |
| SlWRKY30IIc-target 1  off-target site 3 | For | AGAGAATCATTGTGCTTCTTCA |
|  | Rev | CCCCATTGAGTCCCTTCTTCATA |
| SlWRKY30IIc-target 2  off-target site 1 | For | ACGACTCTAAGAGAGGGACTGA |
|  | Rev | CATTGAGTCGGATTGTTTAC |
| SlWRKY30IIc-target 2  off-target site 2 | For | TACGCCATCTACCCCTAATTG |
|  | Rev | GGTAGTCACCACAATGCTTGG |
| SlWRKY30IIc-target 2  off-target site 3 | For | TATTGTACTAAAGCACTTTCTACA |
|  | Rev | GATCAGGTTGGATTCACCTCCTCA |

**Table S6. Primers used for qRT-PCR analysis.**

| SlJAZ1-qPCR-F | TTCCCTCAAGGTGGAATGAAGGCT |
| --- | --- |
| SlJAZ1-qPCR-R | TCCGAAACTCGGAACCACCAAATC |
| SlJAZ2-qPCR-F | ACCTGATCAACCAGAGAAGGCA |
| SlJAZ2-qPCR-R | AAACTCACACCAGATTGATCAGCTGT |
| SlJAZ3-qPCR-F | TTCCCTGCTGACAAAGCTAGAGCA |
| SlJAZ3-qPCR-R | AGGGTGCAGATGAAACTGATCCGA |
| SlJAZ4-qPCR-F | GCCAAAGCCTCAGCAACAAAGGAT |
| SlJAZ4-qPCR-R | ATCACTGCTCTGGCTTTCTCTGCT |
| SlJAZ5-qPCR-F | TCAGCTGTTCCGTCTAGCAGCATT |
| SlJAZ5-qPCR-R | TGCATTTGGTGTAACAGGTGGTGC |
| SlJAZ6-qPCR-F | AGTCGATGCTGGTCTCAAACGTCA |
| SlJAZ6-qPCR-R | TCGAAGACATTGACCATCCCACCA |
| SlJAZ7-qPCR-F | TTGCTATGGCTCGTAGAGCAACTC |
| SlJAZ7-qPCR-R | TTTCCCAATGAACGCTTGACGACG |
| SlJAZ8-qPCR-F | TCGTCAACCTCCCAATCATAAC |
| SlJAZ8-qPCR-R | GGAAAGGGTAGTGAGTGCATC |
| SlJAZ9-qPCR-F | TTTGGAGCTCACTCTTATGCCTCC |
| SlJAZ9-qPCR-R | AGCTCAGTAGCATCGGAAACCACA |
| SlJAZ10-qPCR-F | GGAACTCACTCTTTCTCCTAGCAAC |
| SlJAZ10-qPCR-R | TGGTGATGAAGGCTCAGACAGCTT |
| SlJAZ11-qPCR-F | GGAGTTTAGGCTTATGCCACCTTC |
| SlJAZ11-qPCR-R | GGCTCAGATATTGGTGACAGACTC |
| SlJAZ12-qPCR-F | TGCGCATTCCGAGGCATGATGATA |
| SlJAZ12-qPCR-R | CCTTCTTGCAATTGGCAACTCTGCT |
| SlMYC2-qPCR-F | AGCAGGAGCATCGGAAGAA |
| SlMYC2-qPCR-R | CCAAATCGGGCTGGAACTA |
| SlWRKY30IIc-qPCR-F | GGTCAGAAGGCAGTGAAGAACAG |
| SlWRKY30IIc-qPCR-R | GTGATCACAATTGATGGGTCTTC |
| SlVQ15-qPCR-F | GTAGTTAGAGCTCCAGATCACC |
| SlVQ15-qPCR-R | TTAATACGGTAGTTGGGGTACG |
| SlPDF-qPCR-F | GCAAAGCACCAAGCCAAAC |
| SlPDF-qPCR-R | GCATAGACACTTCCTTTGG |
| SlPI-2-qPCR-F | CCTATTCAAGATGTCCCCGTTC |
| SlPI-2-qPCR-R | GGGCAATCCAGAAGATGG |
| pSlWRKY30IIc-1-qPCR-F | GAAGTCAAGTTTCAATAATTTTA |
| pSlWRKY30IIc-1-qPCR-R | TTAAAAGTTATTAGTCCTCTAAAG |
| pSlWRKY30IIc-2-qPCR-F | TAGATTTCTTAATTCTTACTCCA |
| pSlWRKY30IIc-2-qPCR-R | CAATAGGGAAATACACTTACAAA |
| pSlVQ15-1-qPCR-F | CCCGGTTGAATTGTCAATGTATA |
| PSlVQ15-1-qPCR-R | TTCTTACTCTGCCTAATTTCCGC |
| pSlJAZ3-1-qPCR-F | TAGAGAGAGTTGTTTATTACGA |
| pSlJAZ3-1-qPCR-R | AAAAGATGGTTAACATTGGCA |
| pSlJAZ3-2-qPCR-F | AAGTGTGTTACTCAAAGGCTAAA |
| pSlJAZ3-2-qPCR-R | CTGCGGCTACAGAGAAAACG |
| pSlJAZ7-1-qPCR-F | TGCGAATATTTTCAGCCCCC |
| pSlJAZ7-1-qPCR-R | GAGCGAACTAGCCTCACCTG |
| pSlJAZ9-1-qPCR-F | ATTAGCTAACAATATAACAGAAAGT |
| pSlJAZ9-1-qPCR-R | ACGCTGGATCCTATTACTGA |
| pSlJAZ11-1-qPCR-F | TTCTACTGCTCCTTATTTTCA |
| pSlJAZ11-1-qPCR-R | CTTTGACGTGAGAGATTAAGT |
| pSlActin2-qPCR-F | CGAGAAGTACGTGATGCAGTGT |
| pSlActin2-qPCR-R | CCAGTCCAGATACCTAGTCAGC |
| SlActin2-qPCR-F | TTGCTGACCGTATGAGCAAG |
| SlActin2-qPCR-R | GGACAATGGATGGACCAGAC |
| SlVQ1-qPCR-F | AAGACAAGTGCCTCGAGATTTA |
| SlVQ1-qPCR-R | TGGTGATGACGATTTTACTGGA |
| SlVQ2-qPCR-F | AGAGCTTCCAAGAAAACACCTA |
| SlVQ2-qPCR-R | TTGATTGGACAAGTGTGACAAC |
| SlVQ3-qPCR-F | ATCGCTCCATCGCCACCAATTC |
| SlVQ3-qPCR-R | CACCACTGAGGAGGAAGGAGGAAG |
| SlVQ4-qPCR-F | AGAAAACTCCAACGACTCATCT |
| SlVQ4-qPCR-R | TATTGGACCTTTATGAGCTCCC |
| SlVQ5-qPCR-F | CGAGTTAACAAGCCCTAAAACC |
| SlVQ5-qPCR-R | AATTCGTGATGCTAGCGTTTAC |
| SlVQ6-qPCR-F | GCTTCGTCTTTCAAACAGGTAG |
| SlVQ6-qPCR-R | TTAATTGGAGGGATCGGATTCC |
| SlVQ7-qPCR-F | CATGCTTAACAATGGAGGTAGC |
| SlVQ7-qPCR-R | GGCTTTTTCTTCTTCCTCCATC |
| SlVQ8-qPCR-F | CTCACTGGTCTTTCGAGTCATA |
| SlVQ8-qPCR-R | ATCAAAAATGGCAGCTGATGAG |
| SlVQ9-qPCR-F | CCAACTACTGTGTTGACAACAG |
| SlVQ9-qPCR-R | GCAACAGAATTAGGCACAGTAG |
| SlVQ10-qPCR-F | GACATTTGGATTCACTTGGACC |
| SlVQ10-qPCR-R | CCAGGCATTAAAGCATCAATCA |
| SlVQ11-qPCR-F | GATTCTCTACTGGGCTATCACC |
| SlVQ11-qPCR-R | ACAAAGGAGGAGTTTGTTGGAG |
| SlVQ12-qPCR-F | CAACGCCACTCAAATTACGTAA |
| SlVQ12-qPCR-R | TAGGGTTTGCATGGATTACCTT |
| SlVQ13-qPCR-F | GCCTCCGAGTATGAGATTACAA |
| SlVQ13-qPCR-R | GGCGGTCTAGCAATATTGTTTT |
| SlVQ14-qPCR-F | GTCCATGGGAGAAAAAGAATGG |
| SlVQ14-qPCR-R | GTTTTCTTCATCCCAACAAGCT |
